# Nutrition and physical activity interventions delivered to children and young people during cancer treatment: a systematic literature review

**DOI:** 10.1007/s00520-026-11194-9

**Published:** 2026-09-16

**Authors:** Alexis S. Ross, Émilie Bertrand, Marta Seretny, Andrea Braakhuis, Hanna van Waart, Amy L. Lovell

**Affiliations:** 1 Department of Nutrition and Dietetics, Faculty of Medical and Health Sciences, The University of Auckland, Private Bag 92019, Auckland, 1142 New Zealand; 2 Starship Blood and Cancer Centre, Starship Child Health, Health New Zealand, Te Whatu Ora, Auckland, New Zealand; 3 Te Aka Matauranga Matepukupuku (Centre for Cancer Research, University of Auckland), Auckland, New Zealand; 4 Department of Anaesthesiology, Faculty of Medical and Health Sciences, The University of Auckland, Auckland, New Zealand; 5 Department of Anaesthesia, Te Toka Tumai, Te Whatu Ora Health New Zealand, Wellington, New Zealand

**Keywords:** Childhood cancer, Nutrition, Physical activity

## Abstract

**Purpose:**

Evaluate the evidence for nutrition and/or physical activity interventions delivered during cancer treatment and determine their efficacy.

**Methods:**

The protocol was registered on PROSPERO (reference CRD42024513364) and completed according to the PRISMA guidelines. A comprehensive search of MEDLINE, Embase, SCOPUS, and ProQuest Health & Medical Sciences databases was conducted from inception to March 22, 2024. Risk of bias was assessed using ROB1 for randomised controlled trials, the Newcastle-Ottawa Scale for non-randomised studies, and the NIH tool for uncontrolled studies.

**Results:**

Of the 8349 articles identified, 77 were eligible for inclusion and data extraction. These included 4 nutrition-only, 68 physical activity-only, and 4 combined nutrition and physical activity interventions. Due to significant heterogeneity in study design, variables, and outcomes, a meta-analysis was not performed. There was consistent evidence that physical activity interventions can improve exercise capacity and strength in the paediatric cancer population while benefits to physical function are inconsistent. Effective interventions utilised supervised physical activity of moderate intensity. Emerging evidence suggests that nutrition interventions can improve diet quality.

**Conclusion:**

Future prospective intervention studies should consider both nutrition and physical activity and incorporate standardised outcome measures to maximise advancements in this field.

**Supplementary Information:**

The online version contains supplementary material available at https://doi.org/10.1007/s00520-026-11194-9.

## Introduction

Childhood cancer remains a leading cause of childhood death globally, though 5-year survival rates now exceed 85% in high-income countries [1]. Children with cancer experience morbidity not only due to their cancer but also its treatment and hospitalisation, which negatively impact their quality of life [2, 3], nutritional status [4–8], and physical function [9, 10]. The indirect effect of cancer treatment contributes to poor health behaviours such as low fruit and vegetable intake [11] and reduced physical activity [12–15], which can persist into survivorship [16–18]. Consequently, survivors are at increased risk of long-term health complications including obesity [19–21], metabolic syndrome [22–24], cardiovascular disease [25], and exercise intolerance [26]. These ongoing sequelae underscore the importance of addressing nutrition and physical activity during treatment to prevent complications and improve long-term health outcomes.

Children undergoing cancer treatment often consume diets that are adequate in quantity but poor in quality, characterised by low fruit and vegetable intake, excessive sodium and sugar consumption, and inadequate dairy consumption [11, 27]. This dietary imbalance can develop as a consequence of treatment side effects such as taste changes and altered food preferences [28], and changes in parenting style towards more permissive food choices [29], while the constraints of the hospital environment, chemotherapy side effects, and cancer-related fatigue decrease activity levels [12, 13]. This contributes to muscle atrophy, a decline in strength, and reduced energy expenditure, creating a self-perpetuating cycle of inactivity [30]. Consequently, the combination of reduced physical activity and poor diet quality contributes to the increased prevalence of malnutrition and obesity as children progress through treatment.

Malnutrition, defined as undernutrition (BMI < 5th percentile) or overnutrition (BMI ≥ 85th percentile) [31, 32], affects up to 90% of children receiving cancer treatment [7, 33]. By treatment completion approximately 40% of children are overweight or obese compared to 15% at diagnosis [6, 8, 34]. This negatively impacts treatment efficacy via decreased chemotherapy tolerance, altered pharmacokinetics, treatment delays, infections, deconditioning, reduced immunity, and decreased quality of life [6, 33–35]. Despite these well-documented associations, supportive care addressing nutrition and physical activity across the childhood cancer continuum remains limited [36, 37].

This systematic review evaluated the existing evidence for the implementation of nutrition and/or physical activity interventions during active treatment in children and young people with cancer and determined their efficacy to inform future intervention-focused research.

## Methods

A search of PROSPERO, MEDLINE, Cochrane Database of Systematic Reviews, and JBI Evidence Synthesis was performed (29/11/2023); no current or in-progress systematic reviews on the topic were identified. This systematic review was conducted in accordance with the Preferred Reporting Items for Systematic Reviews and the Meta-Analyses (PRISMA) statement (2020) [38] and in accordance with an a priori protocol registered through PROSPERO (CRD42024513364) [39].

### Search strategy

The search strategy, including all identified keywords and database-specific MeSH terms, was developed with a library specialist and adapted for each of the four databases: MEDLINE (Ovid Web 1966–2024), Embase (1980–2024 March 22), SCOPUS, and ProQuest Health & Medical Sciences in March 2024). The inclusion and exclusion criteria were developed a priori and refined during the pilot process, where four reviewers (AR, ALL, EB, HvW) independently screened a sample of 50 studies. Conflicts were resolved through discussion, leading to modifications of the criteria. No limits were applied to publication dates.

#### Study selection

All records identified were uploaded into the Covidence systematic review software (Veritas Health Innovation, Melbourne, Australia). Duplicate articles were removed by the software and manually scanned. Inclusion criteria followed the PICO framework: (P) Population of children and young people 0–21 years (≥ 75% of participants) diagnosed with childhood cancer; (I) Intervention of nutrition (dietary) and/or physical activity, initiated between diagnosis and treatment completion or before day zero of a haematopoietic stem cell transplant; (C) Comparators were as defined by the studies such as a control group or pre- and post-intervention; (O) The primary outcomes were the change in dietary intake for nutrition interventions and impact on physical function for physical activity interventions.

Four reviewers screened titles and abstracts (AR, EB, HvW, and ALL), and full-text eligibility was assessed by the same team with at least two reviewers screening each record. Conflicts were resolved by discussion and consensus between at least three reviewers. Studies that included the same participants but reported on different outcomes were collated and referred to as one intervention.

### Data extraction

Data were extracted by two reviewers (AR and EB). Primary outcomes included physical function and diet quality as reported by the author. Secondary outcomes included anthropometry, body composition, dietary intake (calories, macronutrients), physical activity level, cardiorespiratory fitness, fatigue, quality of life, feasibility, and adverse events. Feasibility variables included recruitment: the number of participants recruited out of those that were eligible and approached; retention: participants that remained in the study; adherence: attendance at physical activity and nutrition sessions or activities; and compliance: completion of the intervention specifications.

### Evidence synthesis

Study findings were summarised narratively, as a meta-analysis could not be performed due to heterogeneity in outcome measures. Vote counting based on the direction of effect was used to synthesise findings [40]. Outcomes were categorised as diet quality, anthropometry and body composition, physical function, exercise capacity, physical activity, fatigue, and quality of life.

Effect direction in studies with controls was determined using baseline adjusted between-group intervention effect where reported, or mean change (pre-post) between groups. Cohort studies effect direction was determined by mean change (pre-post) intervention. Studies with multiple measures within an outcome domain had effect direction reported if > 70% of results favoured the same direction. Sign test was calculated in GraphPad (https://www.graphpad.com/quickcalcs/binomial1/).

### Risk of bias assessment

Quality assessment was conducted by one reviewer (AR) using the Cochrane Risks of Bias tool (ROB-1) [41] for randomised controlled trials (RCTs), the Newcastle-Ottawa Scale [42] for non-RCTs, and the NIH cohort studies tool [43] for uncontrolled studies.

## Results

### Study selection and characteristics

**Fig. 1:**
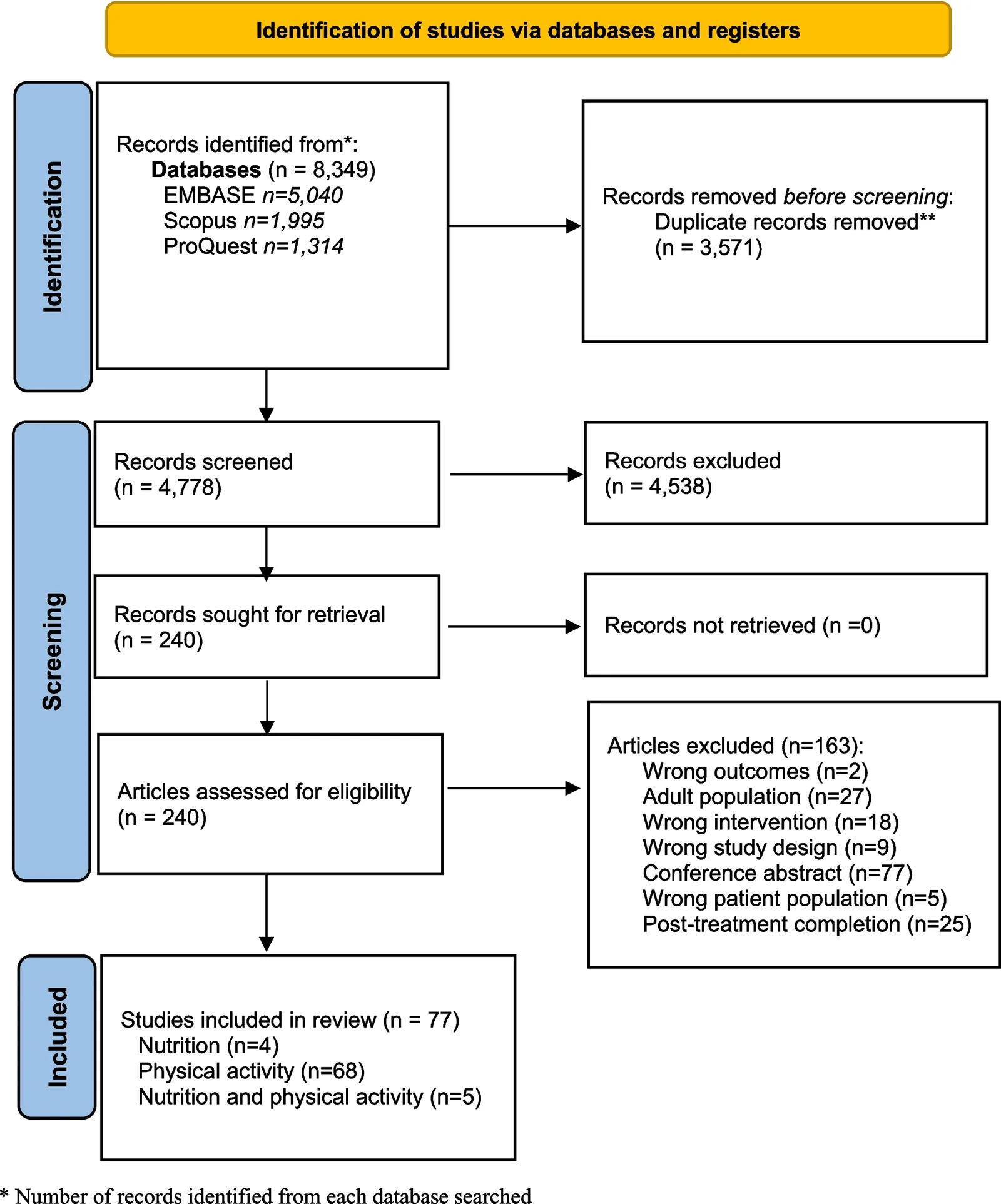
PRISMA flowchart for the selection of the original studies on nutrition and physical activity interventions delivered to children and young people during cancer treatment. *Number of records identified from each database searched. **Duplicates excluded using Covidence Software

The search identified 8349 studies, and 77 articles (reporting on 66 original cohorts) were selected for inclusion in this systematic review (Fig. 1). Among these, four studies delivered a nutrition intervention [44–47], while another four studies, represented across five articles, combined nutrition and physical activity interventions [48–52] (Table 1). The remaining 68 articles, covering 58 original cohorts, focused solely on physical activity interventions.

**Table 1:** Summary of study characteristics

Characteristic	Physical activity	Physical activity and nutrition	Nutrition
	*n*	*n*	*n*
Interventions	58	4	4
Papers	68	5	4
Intervention participants	1618	97	80
Cohort size			
1– 9	7	1	
10– 19	24	1	2
20– 49	19	1	2
50– 99	6	1	
≥100	2		
Age (mean or median)			
≤ 4 years	3		
5 to 9 years	15	3	2
10 to 14 years	29		
15 to 21 years	3	1	
Not reported	8		2
Diagnosis			
ALL	14	3	3
Bone tumour	3		
Mixed	37	1	1
Brain tumour	1		
Not reported	3		
Timing of intervention			
0–4 weeks	14	1	1
1–3 months	3	1	
Maintenance	6	2	2
HSCT	9		
Other	26		1
Duration			
1 session	3		
< 2 weeks	2		
2–4 weeks	12	1	
> 1–3 months	15	1	1
> 3–6 months	10		2
> 6–12 months	5	2	1
> 1 year	3		
Individualised	8		

*ALL* Acute lymphoblastic leukaemia, *HSCT* Hematopoietic stem cell transplantation, *Other* not limited to stage of treatment, *Individualised* duration based on patient needs or treatment duration

### Nutrition interventions

#### Study characteristics

Of the nutrition interventions, two were RCTs [47, 51], and two used historical control groups [44, 49]. Sample sizes for intervention participants ranged from 6 [51] to 61 [48] (Supplementary Table S1). Six studies only included children diagnosed with acute lymphoblastic leukaemia (ALL) [44, 46, 47, 49–51], while two studies involved participants with mixed cancer diagnoses [45, 48] (Table 1).

#### Intervention design and delivery

Half of the interventions (*n* = 4 studies) were initiated during maintenance treatment for ALL [44, 47, 50, 51]: two during induction treatment [46, 49], and one during early treatment (4–12 weeks after diagnosis) [48, 52].

The duration of interventions ranged from 4 weeks [49] to 12 months [47, 48, 51]. Interventions included one-on-one nutrition counselling sessions with a registered dietitian [46–49, 51], nutrition education workshops [45], and a self-guided online programme with support from a lifestyle coach [50] (Supplementary Table S2).

#### Assessment tools/measures

Dietary intake was measured using 24-h diet recall [45–48, 50] and food frequency questionnaires [45]. Reported data included diet quality [48, 50], energy (calories) [45–48, 50, 51], and macronutrients [45, 46, 48, 50]. Consumption of foods or food groups included fruit and vegetables [45, 46, 50], grains [45, 46, 50], and dairy [45, 46, 50], protein-containing foods [46], meat or animal proteins [45, 50], legumes [45, 50], sugar or added sugar [46, 50], and sodium [50, 52].

Anthropometry was reported primarily as body mass index (BMI) z-scores [44, 48, 50, 51] or BMI percentile [47], using age- and sex-adjusted growth charts from the World Health Organization [53] or the Centers for Disease Control and Prevention [54].

#### Outcomes

##### Dietary intake

Diet quality improvement was inconsistent across the two studies that reported this outcome. After a 3-month intervention, the mean Healthy Eating Index (HEI) [55] score increased from 44.5 (9.08 SD) to 47.7 (7.91 SD) out of 100 (mean change 3.14, 95% CI −3.55 to 9.82; *p* = 0.29) [50]. A 12-month intervention reported an increase in mean Diet Quality Index (DQI) of 5.22 (9.95 SD; *p* = 0.003) from the baseline mean of 48.53 (10.4 SD) out of 100, while the Healthy Diet Index (HDI) score did not change significantly (*p* = 0.065) [48]. The consumption of fruit and vegetables increased following nutrition education workshops [45] and nutrition counselling during intensive treatment in children with ALL [46]. Fruit and vegetable intake did not change substantially following a 3-month self-lead online education programme [50]. Increases in calorie intake were reported by two studies over the first 4 weeks (*p* = 0.0002) [46] and 12 months (*p* = 0.033) [48] of treatment (Supplementary Table S5). When expressed as a percentage of energy intake, two studies found an increase in protein intake at 3-month (*p* = 0.04) [50] and 6-month (*p* = 0.027) [46] post-intervention. Effect direction for dietary intake is summarised in Table 2.

**Table 2:** Effect direction of nutrition intervention outcomes

Study	Study design	*n*	Diet quality	Fruit and vegetables	Dairy	Sodium	Sugar	Anthropometry and body composition	Physical activity
Walters (2021) [46]	CS	23		▲	▼		▲	▲	
Viscardi (2021) [45]	CS	12		▲	◄►		▲		
Napartuk (2023) [48, 52]	CS	36	▲			▲		▼	
Zhang (2019) [50]	CS	13	▲	◄►	▲	▼	▲	▼	▼
Orgel (2021) [49]	NRCT	40						◄►	
Hill (2018) [44]	NRCT	33						▲	
Li (2016) [47]	RCT	12						▼	
Moyer-Mileur (2009) [51]	RCT	6						◄►	◄►

*CS* cohort study, *NRCT* non-randomised control trial, *RCT* randomised control trial, *n* number of participants in intervention group/cohort; ▲: direction of positive health impact; ◄►: no change or unable to determine health impact; ▼: direction of negative health impact

### Physical activity interventions

#### Study characteristics

Among the 62 physical activity interventions included in this review (reported across 73 articles), the majority (*n* = 38) recruited participants with mixed cancer diagnoses, while 17 studies only included children with ALL (Table 1). Twenty studies were RCTs [51, 56–74], 11 quasi-controlled trial designs [75–85], and 5 comparing to historical control groups [49, 86–89] (Supplementary Table S1). Most intervention groups consisted of 10 to 50 participants, while eight studies had fewer than 10 participants [51, 74, 82, 89–93].

Timing of intervention delivery varied with 26 studies not targeted towards a specific stage of treatment. Nine interventions started upon hospital admission for conditioning treatment prior to a haematopoietic stem cell transplant [71–74, 84, 85, 89, 94, 95].

#### Intervention design and delivery

Interventions were predominantly delivered in inpatient hospital settings (*n* = 24) and supervised individually by healthcare professionals (Supplementary Table S4). Other settings included outpatient clinics (*n* = 7), participants’ homes (*n* = 9), or a combination of these locations (*n* = 15). The most common types of physical activity intervention were aerobic, resistance, flexibility training, and balance exercises. Physical activity was primarily facilitated through structured training programmes, with a smaller number using yoga and exergaming (i.e. technology-driven physical activity). The frequency of physical activity ranged from one session every 2 weeks to twice daily, with three to five sessions per week being the most common. Sessions typically lasted 30 to 60 minutes and were conducted at low to moderate intensity.

#### Assessment tools/measures

Physical function was evaluated using the Timed-Up-and-Go [63, 80, 85, 87, 91, 92, 96–99], Timed-Up-and-Down-Stairs [60, 63, 91, 97, 99], Sit-to-Stand [80, 96, 98], rise from floor [98, 100], and floor stand based on time [98] (Supplementary Table S3). Balance was assessed using the Flamingo Test or single leg balance duration [69, 80, 94, 96]. Flexibility was measured with the sit and reach test [51, 69, 85, 92, 101, 102]. Exercise capacity and endurance were assessed using the 6-minute walk test (6MWT) [56, 61, 69, 70, 73, 82, 94, 98, 101–103] and the 9-minute walk run test [60, 87]. Cardiopulmonary fitness was assessed using maximal [67, 73, 80, 96, 99, 101, 104] and peak oxygen uptake [63, 67, 96], maximum watts [80, 104], and ventilatory threshold [63, 73].

Strength was assessed with hand-grip strength [68, 73, 80, 94, 96], dynamometry knee extension [60, 73, 85, 94, 102], and repetition maximum for bench press [63, 91, 97, 99], lateral row [63, 91, 97, 99], and leg press [63, 91, 97, 99].

Twenty studies assessed physical activity levels with wearables recording steps, gait cycles, time and/or intensity with Actigraphy [50, 63, 64, 79, 83, 88], Fitbits [77, 98, 105], and pedometers [51]. Ten studies [61, 63, 68, 70, 81, 88, 92, 101, 106, 107] used questionnaires, most commonly The Godin-Leisure-Time Exercise Questionnaire [61, 65, 88, 92, 101].

Fatigue was measured using the Paediatric Quality of Life Multidimensional Fatigue Scale [61, 65, 70–72, 75, 85, 90, 94, 95, 108] and the Fatigue Scale [66, 68, 88, 105]. Quality of life was assessed using the Kinder Lebensqualität questionnaire (KINDL) [70, 73, 77] and Paediatric Quality of Life [60, 63, 68, 69, 71, 74, 82, 92, 94, 98, 101, 103, 109].

Anthropometry was assessed using BMI as kg m^−2^ [69, 70, 89], age percentile [56, 102], and z-scores [57]. Body composition was reported as body fat, lean body mass/fat-free mass, and bone density, using DEXA [57, 62], bioelectric impedance analysis [69], or estimation using skinfold measures [89].

## Outcomes

### Physical function

Physical function results favoured intervention effect in 12 of 16 interventions, with an additional 5 interventions showing unclear effect direction (Table 3). In two RCTs, no statistically significant difference was found in Timed-Up-and-Go [63] or Timed-Up-and-Down-Stairs [60, 63]. Both utilised aerobic and resistance training, with one delivering supervised sessions three times a week for 19 weeks [63], and the other five supervised sessions over 4 months in addition to home-based exercises 3–5 days a week [60]. The non-RCT found no difference in Timed-Up-and-Go between groups after a 6-month supervised intervention delivered exclusively during hospital admissions [80]. The three small cohort studies showed improvement in Timed-Up-and-Go after 12 weeks twice-weekly yoga [92] and after 8 [91] and 16 weeks [97] of supervised aerobic, flexibility, and resistance training three times a week in children with ALL. Rise from floor increased following 8 weeks of aerobic, resistance, flexibility, and balance training three times a week [103]. Improvements in balance were reported in two studies [69, 96]. Flexibility improved in two interventions: one involving twice-weekly yoga classes for 12 weeks [92] and another using a combination of resistance, aerobic, balance, and flexibility training two to three times a week for 6 months [69].

**Table 3:** Effect direction plot of outcomes from physical activity and nutrition interventions

Study	Study design	*n*	Diet quality	Anthropometry and body composition	Exercise capacity	Physical function	Muscle strength	Physical activity	Fatigue	Quality of life
Atkinson (2023) [101]	CS	20			▲	▲	▲	▲	▲	▲
Bogg (2015) [94]	CS	14			▼	▼	▼	▲	▼	▲
Caru (2020) [106]	CS	16						▲		
Chamorro-Vina (2010) [89]	CS	7		▲						
Dalla Santa (2023) [103]	CS	14			▲	▼				▲
Diorio (2015) [95]	CS	11							◄►	◄►
Esbenshade (2014) [102]	CS	12		▲	▲	▲	▲			
Govardhan (2019) [107]	CS	18						▲	▲	
Grimshaw (2024) [98]	CS	20			▲	▲		▲		▲
Hooke (2016) [105]	CS	16						▲	◄►	
Napartuk (2023) [48]	CS	36	▲	▼						
Orsey (2017) [109]	CS	10								▲
Platschek (2017) [90]	CS	9							▲	
San Juan (2007) [91, 97]	CS	7			▲	▲	▲			
Stein (2019) [108]	CS	10							◄►	
Tanner (2017) [100]	CS	135				▲				
Wurz (2014) [92]	CS	8				▲		▲		▲
Zhang (2019) [50]	CS	15	▲	▼				◄►		
Corr (2017) [87]	NRCT	14			▲	▼				
Götte (2018) [77]	NRCT	21				◄►	▲	▲		▲
Hayati (2019) [78]	NRCT	32							▲	
Hooke (2019) [88]	NRCT	29						▼	▲	
Müller (2014) [79]	NRCT	10		▲				▲		
Nielsen (2020) [80, 96, 104, 131]	NRCT	120			▲	▲	▲			
Orgel (2021) [49]	NRCT	40		◄►						
Ouyang (2019) [81]	NRCT	57						▲	▲	
Rossi (2017) [85]	NRCT	36				▲	◄►		▲	
Sonoda (2024) [82]	NRCT	7			▲					▲
Winter (2013) [83]	NRCT	16						▲		
Wright (2003) [86]	NRCT	40				▲				
Yeh (2011) [75]	NRCT	12							▲	
Cox (2018) [56]	RCT	53		▼	▼	▼	◄►	▼		
Fiuza-Luces (2017) [63, 99, 132]	RCT	24		◄►	▲	▲	▲	▼		▲
Gaser (2022) [64]	RCT	21				◄►		▲		
Hamari (2012) [65]	RCT	17				◄►		◄►	◄►	
Hartman (2009) [57]	RCT	25		◄►		◄►				
Hinds (2007) [66]	RCT	14							◄►	
Huang (2023) [71]	RCT	23							▲	▲
Kaushal (2013) [58]	RCT	36							▼	
Khodashenas (2017) [59]	RCT	10								◄►
Kowaluk (2022) [67]	RCT	10			▲					
Lam (2018) [68]	RCT	37					▲	▲	▲	▲
Marchese (2004) [60]	RCT	13			▲	▲	▲			▲
Masoud (2023) [61]	RCT	22			▲			▲		
Moyer-Mileur (2009) [51]	RCT	6		◄►		◄►		◄►		
Saultier (2021) [69]	RCT	41		▲	▲	▲	▲		▲	▲
Schechter (2023) [133]	RCT	62							▲	
Senn-Malashonak (2019) [73, 134]	RCT	35			▲		▲			◄►
Stössel (2020) [70]	RCT	16			▲		▲	▲	▲	▲
Waked (2018) [62]	RCT	23		▲						
Sign test *p* value			0.5	0.4531	0.0023	0.0658	0.0063	0.0072	0.0074	0.0001

*CS* cohort study, *NRCT* non-randomised control trial, *RCT* randomised control trial; ▲: direction of positive health impact; ◄►: no change or unable to determine health impact; ▼: direction of negative health impact; n: number of participants in intervention group/cohort

#### Exercise capacity

The direction of effect was positive for 15 of 17 interventions that reported an outcome within the exercise capacity domain (Table 3). The sign test favoured the interventions having a positive effect, being greater than chance (*p* = 0.0023).

Functional capacity (6MWT) improved in two [101, 103] of five cohort studies [94, 98, 101–103], of which two [98, 102] did not report significance due to their feasibility design. 6MWT distance improved after 6 weeks of advanced robotic gait training [101] and after 8 weeks of supervised physiotherapy sessions on top of independent or parent supervised home-plan exercises [103]. Four out of five RCTs [61, 69, 70, 73, 110] found an improvement after aerobic exergaming [61] and aerobic and resistance training [69, 70, 73].

Cardiorespiratory fitness improved in three out of five interventions reported in eight articles [63, 67, 73, 80, 96, 99, 101, 104] (Supplementary Table S7). Three interventions showed an increase in maximal oxygen uptake. One cohort used robotic gait training twice a week for 6 weeks [101], one RCT using aerobic activity video games three times a week for 4 weeks [67], and one intervention that combined social support with supervised aerobic, resistance, and balance exercises during hospital admissions over 6 months [80].

#### Muscle strength

Strength had a positive effect direction reported in 11 of 12 interventions and a sign test *p*-value of 0.0063. Interventions, across four articles, improved lateral row, bench, and leg press repetition maximum after exercise interventions [63, 91, 97, 99]. Effects on hand-grip strength and knee extension were mixed. The interventions that had statistically significant increases in strength all included supervised exercise sessions [60, 63, 69, 101].

#### Physical activity

Physical activity significantly increased in three [92, 106, 107] of six cohort studies [50, 92, 101, 105–107], two RCTs [68, 70], and one non-RCT [81]. Interventions included telehealth-supervised [106], in-person supervised physical activity sessions [68, 70, 81], and supervised yoga sessions [92].

#### Other measures

Anthropometry and body composition changes were not statistically significant in most nutrition [44, 47, 50, 51] and physical activity [56, 57, 63, 69] interventions (Supplementary Table S6). Fatigue results favoured the intervention in 13 of 15 studies with effect direction (sign test *p* = 0.0074). Statistically significant improvement was reported in three [90, 101, 107] of seven [85, 88, 90, 94, 101, 105, 107] cohort studies and in five [58, 61, 68, 70, 71] of eight [58, 61, 65, 66, 68, 70–72] RCTs (Supplementary Table S9). Quality of life scores favoured intervention (sign test *p* = 0.0001) and significantly improved in four [92, 101, 103, 109] of five cohort studies [92, 94, 101, 103, 109]. Of the RCTs, three [68–70] of eight studies [60, 63, 68–71, 73, 82] reported differences in quality of life, along with one non-RCT [77] (Supplementary Table S9).

#### Feasibility

Recruitment rates were reported for 50 interventions, ranging from 42.3 to 100% in exercise interventions and 50–90.9% in nutrition interventions (Supplementary Table S10). Adherence was > 75% for 18 of the 42 interventions which reported this outcome [56, 61, 66–68, 70, 79, 82, 89, 91, 93, 95, 97, 98, 101–103, 106, 107, 111]. Adverse events were uncommon in those who reported on their occurrence.

#### Quality assessment

Sequence generation and allocation concealment had a low risk of bias in most studies which reported their methods with sufficient detail (Tables 4, 5, and 6). Outcome assessors were reported to be unblinded in studies that described this. Incomplete outcome data, due to poor attrition and adherence to assessments, was common.

## Discussion

This review synthesised the evidence for nutrition and physical activity interventions delivered to children and adolescents during cancer treatment. While physical activity interventions demonstrated largely consistent benefits across functional capacity, muscle strength, physical activity, fatigue, and quality of life, the evidence for nutrition interventions was limited and heterogenous. Variation in study design, intervention characteristics, and outcomes precluded meta-analysis. Despite this, three key messages emerge from this review: (1) physical activity interventions appear beneficial during treatment, although optimal intervention design remains unclear; (2) nutrition interventions are feasible, but evidence for their effectiveness is limited and inconsistent; and (3) nutrition and physical activity are physiologically and behaviourally interconnected and should not be considered in isolation.

### Physical activity interventions demonstrate consistent benefits, but intervention heterogeneity prevents identification of ‘best practice’

The strongest evidence identified in this review relates to physical activity interventions. Across studies, the overall effect direction consistently favoured the intervention for functional capacity, muscle strength, physical activity participation, fatigue, and quality of life, suggesting that appropriately designed physical activity interventions can be safely implemented during active cancer treatment, are a valuable addition to supportive care, and provide meaningful benefits.

Findings for physical function were less consistent, reflecting the multifactorial nature of functional impairment during treatment. Functional tests require a combination of coordination, balance, muscle strength, speed, and neuromuscular control [112, 113]. Thus, improvements in individual domains may not translate into measurable changes. Treatment-related toxicities including peripheral neuropathy, fatigue, and inactivity may further attenuate improvements.

Interventions that incorporated resistance training and supervision appeared beneficial in preserving overall muscle strength and function during treatment, while supervision may improve adherence, confidence, and adaption based on symptoms.

Despite these findings, substantial heterogeneity in interventions, including timing, frequency, and intensity, and outcome measures limits our ability to determine the optimal exercise prescription for children undergoing treatment. Nevertheless, intervention effectiveness appeared to be influenced by several modifiable characteristics, including supervision, exercise modality, behavioural support, and intervention timing. Although current paediatric guidelines recommend at least 60 minutes of moderate-to-vigorous activity per day [114, 115], many children undergoing cancer treatment experience substantial barriers to participation. The positive effects observed across interventions suggest that clinically meaningful benefits can still be achieved through flexible, treatment-adapted programmes, even when guideline targets may not be attainable [116]. Future research should therefore focus not only on whether physical activity is beneficial, but also on identifying which intervention components are the most effective, for which patients, and at what stage of treatment.

### Nutrition interventions appear feasible, but evidence for effectiveness remains underdeveloped

Nutrition interventions demonstrated that positive changes to dietary intake can be achieved during treatment; however, the evidence base remains limited and inconsistent. Substantial heterogeneity in intervention design, outcome measures, and dietary assessment methods prevented meaningful synthesis of findings and limited interpretation of effectiveness.

The limited number of nutrition interventions likely reflects the historical focus on preventing undernutrition during treatment. However, improved survival has increased attention on diet quality, overweight and obesity, and the long-term health behaviours of childhood cancer survivors [117].

Nutritional management during treatment remains inherently complex and inconsistent findings across nutrition interventions may reflect that. Treatment-related factors such as altered taste and smell [28, 118, 119], nausea, mucositis, corticosteroid-induced appetite changes [119, 120], dietary restrictions [121], family feeding practices [122], and psychosocial distress [123, 124] all influence dietary intake and food choices throughout treatment. Consequently, interventions that are effective during one phase of treatment may not equally be effective during another and may differ according to diagnosis and treatment intensity.

Only four interventions reported dietary intake outcomes, and outcome measures varied substantially between studies. Feasibility concerns associated with dietary assessment were noted, with one study modifying data collection approaches to reduce participant burden [48, 52]. Dietary intake was also inconsistently standardised against estimated nutritional requirements, limiting interpretation of reported changes. Future studies should prioritise approaches that balance feasibility, participant burden, and accuracy, while incorporating important contextual factors such as nutrition impact symptoms, nutrition support use, and treatment intensity.

### Nutrition and physical activity are physiologically interconnected but are rarely addressed together

A key finding of this review is the limited integration nutrition and physical activity interventions despite their likely interdependence physiologically and behaviourally. Only four studies evaluated combined nutrition and physical activity interventions, and nutrition and physical activity outcomes were largely reported separately, limiting understanding of potential synergistic effects. This represents an important gap in the current evidence base.

Nutrition and physical activity contribute to many of the same outcomes during treatment, including nutritional status, body composition, muscle mass, functional capacity, appetite, fatigue, and quality of life. Adequate dietary intake provides the energy and protein required for growth, tissue repair, immune function, and preservation of lean body mass, while physical activity provides the anabolic stimulus required to maintain muscle mass, strength, and cardiorespiratory fitness [125, 126]. Consequently, these behaviours should not be considered independently.

Poor diet quality and sedentary behaviour may interact synergistically to accelerate treatment-related declines in health. Inactivity may accelerate deconditioning, declining muscle strength, fatigue, and functional impairment, while inadequate dietary intake may impair recovery and maintenance of lean tissue [127]. Conversely, excessive energy intake in the context of prolonged inactivity may contribute to excess adiposity while simultaneously masking losses in muscle mass, particularly in children receiving corticosteroids [128, 129]. Declining muscle mass and physical function may further contribute to fatigue, reducing participation in physical activity and creating a cycle of progressive deconditioning and functional loss [130]. This bidirectional relationship may help explain why interventions targeting only one aspect of supportive care may have limited impact compared with integrated approaches that simultaneously address nutrition, physical activity, and behavioural factors.

### Feasibility

Despite intervention heterogeneity, nutrition and physical activity interventions were generally feasible, with high participant recruitment and retention rates and few adverse events. Retention and participation appeared higher in supervised interventions, further supporting the potential value of ongoing allied health professional support. Adverse events were rare, reinforcing the safety of these supportive interventions when appropriately delivered. However, adherence and compliance data were inconsistently reported, limiting understanding of dose-response relationship (frequency, intensity, type, duration).

### Strengths and limitations

To our knowledge, this is the first review to synthesise evidence from both nutrition and physical activity interventions delivered during cancer treatment in children and adolescents. Strengths include protocol registration with PROSPERO, a comprehensive search strategy, broad inclusion criteria, and the inclusion of both nutrition-only and physical activity interventions, enabling consideration of these supportive care strategies within a single review.

Several limitations should be considered when interpreting the findings. The evidence base was highly heterogenous with respect to study design, participant characteristics, assessment tools, interventions, and outcome measures. This prevented quantitative synthesis and precluded estimation of pooled effect sizes, limiting our ability to determine the magnitude of intervention effect.

Many studies were also characterised by small sample sizes, which may have reduced statistical power to detect clinically meaningful effects. In addition, the risk of bias in several studies was influenced by the inability to blind participants and study personnel to group allocation. Although this is often unavoidable in nutrition and physical activity interventions, the potential influence on participant behaviour and outcome assessment cannot be excluded.

### Implications and future directions

Consistent with the findings of this review, future supportive care programmes should move beyond isolated nutrition or physical activity interventions and adopt integrated multidisciplinary approaches that recognise the interrelationship between dietary intake, body composition, physical function, fatigue, and quality of life. Interventions should be introduced early following diagnosis to minimise treatment-related declines in nutritional and functional status, whilst maintaining sufficient flexibility to accommodate differing treatment intensities, symptom burden, and family priorities. Physical activity interventions should incorporate supervised aerobic and resistance training where feasible, alongside behavioural strategies and family involvement to support engagement and adherence. Similarly, nutrition interventions should prioritise diet quality and the promotion of healthy dietary behaviours while acknowledging the dynamic and often unpredictable nature of nutritional status during treatment.

Greater standardisation of outcome measures is also needed to facilitate comparisons across studies and support the development of evidence-based supportive care recommendations. Nutrition outcomes should extend beyond anthropometry and include measurements of body composition, diet quality, protein adequacy, and treatment-related nutritional toxicities. Physical activity outcomes should incorporate objective measures of activity participation (e.g. accelerometery), alongside clinically meaningful measures such as exercise capacity, fatigue, and quality of life. Emphasis should be placed on evaluating body composition alongside traditional anthropometric measures, as body weight and BMI alone may not adequately capture treatment-related changes in muscle mass, functional status, or adiposity.

Finally, consistent reporting of intervention adherence, treatment intensity, nutrition support use, and nutrition impact symptoms is required to better understand which intervention components are most effective, for whom, and under what circumstances. As the evidence base evolves, routine supportive care should encourage both healthy dietary intake and safe participation in physical activity throughout treatment, where clinically appropriate, as synergistic components of comprehensive cancer care.

## Conclusion

As it stands, the evidence suggests that physical activity interventions can improve important physical health outcomes during childhood cancer treatment, while nutrition interventions demonstrated feasibility but require further evaluation. The most important gap identified in this review is the lack of integrated interventions that simultaneously address both nutrition and physical activity despite their physiological and behavioural interdependence. Future supportive care research should move beyond single-behaviour interventions and evaluate integrated nutrition and physical activity approaches that use standardised measures and can preserve nutritional status, muscle function, and quality of life throughout cancer treatment.

## Supplementary Information

Below is the link to the electronic supplementary material.

Supplementary file1 (DOCX 269 KB)

## Figures and Tables

**Table 5:**
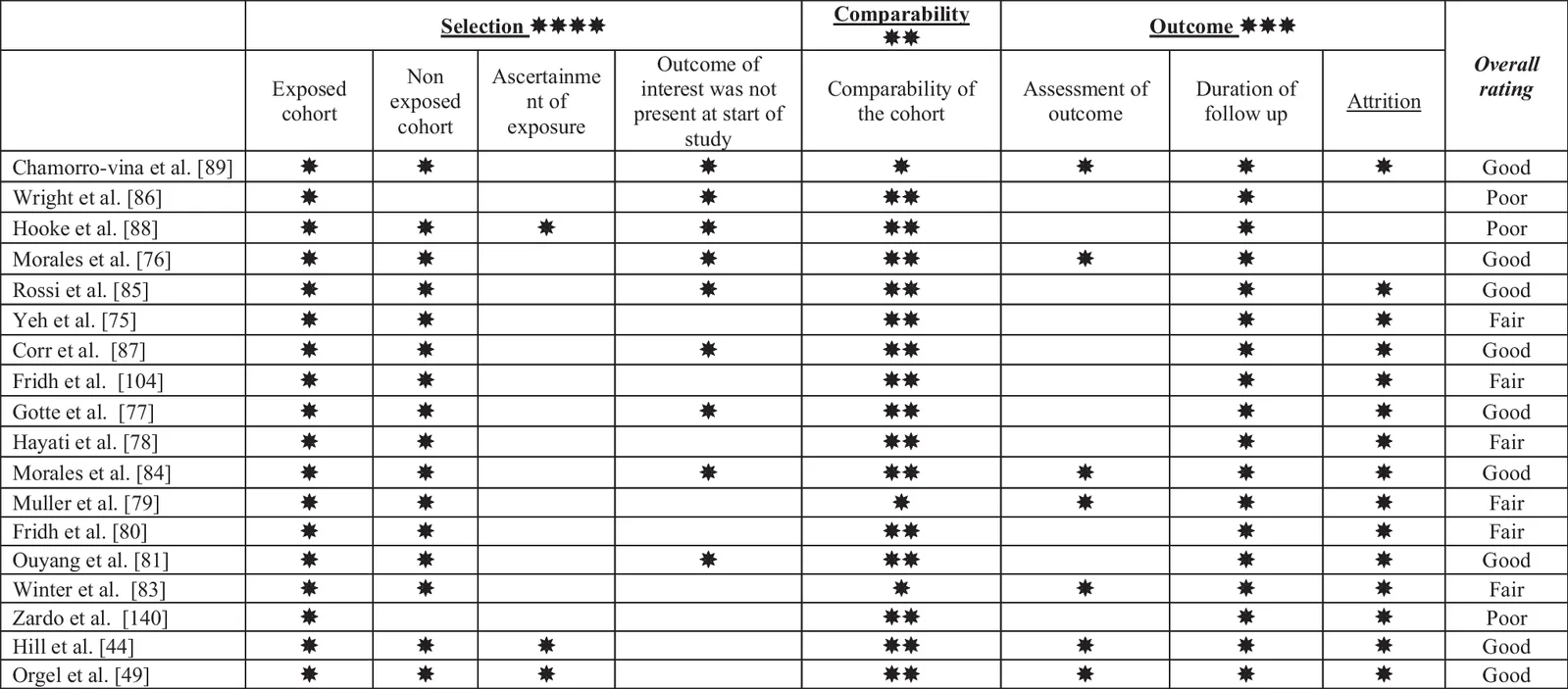
Non-randomised controlled studies quality assessment: Newcastle-Ottawa Scale

## Data Availability

The data from this study is available from the corresponding author upon request.

## Appendix

**Table 4:** Quality assessment of uncontrolled studies: NIH quality assessment tool

	1. Objective	2. Eligibility criteria	3. Cohort	4. Enrolment	5. Sample size	6. Consistent delivery	7. Outcomes	8. Blinded assessment	9. Retention	10. Statistics	11. Interrupted time series
Atkinson et al. [101]	Y	Y	Y	N	Y	Y	Y	?	N	Y	N
Bogg et al. [94]	Y	Y	Y	N	Y	Y	Y	?	Y	Y	N
Caru et al. [106]	Y	Y	Y	Y	Y	Y	Y	?	Y	Y	N
Bluth et al. [135]	N	Y	Y	?	Y	?	Y	?	?	Y	N
Esbenshade et al. [102]	Y	Y	Y	N	Y	Y	Y	?	N	Y	N
Gohar et al. [93]	N	Y	Y	Y	N	?	?	N	Y	N	N
Grimshaw et al. [98]	Y	Y	N	N	Y	Y	Y	N	Y	N	N
Hooke et al. [105]	Y	Y	Y	N	Y	Y	Y	?	Y	Y	Y
Tanner et al. [100]	Y	Y	Y	Y	Y	?	N	N	N	N	N
Tsao et al. [111]	N	Y	Y	Y	Y	Y	Y	N	Y	Y	N
San Juan et al. [91]	Y	Y	Y	?	N	Y	?	N	?	Y	N
San Juan et al. [97]	Y	Y	Y	?	N	Y	?	N	Y	N	N
Dalla Santa et al. [103]	Y	Y	Y	N	Y	Y	Y	N	Y	Y	N
Oschwald et al. [136]	Y	Y	Y	N	Y	Y	Y	N	N	N/A	N/A
Platschek et al. [90]	Y	N	Y	?	Y	N	N	N	Y	N	N
Stein et al. [108]	Y	Y	N	N	Y	N	Y	N	Y	N	N
Taguchi et al. [137]	Y	Y	Y	N/A	Y	N	?	?	N/A	Y	N
Thygeson et al. [138]	Y	Y	Y	N	Y	Y	Y	N	Y	Y	N
Wurz et al. [92]	Y	Y	Y	N	Y	Y	Y	N	N	Y	N
Diorio et al. [95]	Y	Y	Y	N	Y	N	N	N	Y	N	N
Fukuhara et al. [139]	Y	Y	Y	?	Y	?	?	N	?	Y	N
Govardhan et al. [107]	Y	Y	Y	N	Y	Y	N	N	Y	Y	N
Orsey et al. [109]	Y	Y	Y	?	Y	?	Y	N	N	N	N
Napartuk et al. [48]	Y	Y	Y	N	Y	Y	Y	N	N	Y	N
Nielsen et al. [96]	Y	Y	Y	N	Y	N	N	N	Y	N	N
Viscardi et al. [45]	Y	N	?	?	Y	Y	Y	?	?	Y	N
Walters et al. [46]	Y	Y	Y	?	Y	?	Y	?	Y	Y	N
Zhang et al. [50]	Y	Y	Y	?	Y	Y	Y	?	Y	Y	?

*Y* yes, *N* no;?, unclear, *N/A* not applicable

## NIH quality assessment tool for before-after (pre-post) studies with no control group

### Criteria

1. Was the study question or objective clearly stated?
2. Were eligibility/selection criteria for the study population prespecified and clearly described?
3. Were the participants in the study representative of those who would be eligible for the test/service/intervention in the general or clinical population of interest?
4. Were all eligible participants that met the prespecified entry criteria enrolled?
5. Was the sample size sufficiently large to provide confidence in the findings?
6. Was the test/service/intervention clearly described and delivered consistently across the study population?
7. Were the outcome measures prespecified, clearly defined, valid, reliable, and assessed consistently across all study participants?
8. Were the people assessing the outcomes blinded to the participants' exposures/interventions?
9. Was the loss to follow-up after baseline 20% or less? Were those lost to follow-up accounted for in the analysis?
10. Did the statistical methods examine changes in outcome measures from before to after the intervention? Were statistical tests done that provided p values for the pre-to-post changes?
11. Were outcome measures of interest taken multiple times before the intervention and multiple times after the intervention (i.e. did they use an interrupted time-series design)? 

## Newcastle-Ottawa Scale—cohort studies (non-randomised)

Note: A study can be awarded a maximum of one star for each numbered item within the Selection and Outcome categories. A maximum of two stars can be given for Comparability.

## Selection

1. Representativeness of the exposed cohort
  - Truly representative of the average _______________ (describe) in the community
  - Somewhat representative of the average ______________ in the community
  - Selected group of users, e.g. nurses, volunteers
  - No description of the derivation of the cohort
2. Selection of the non-exposed cohort
  - Drawn from the same community as the exposed cohort
  - Drawn from a different source
  - No description of the derivation of the non-exposed cohort
3. Ascertainment of exposure
  - Secure record (e.g. surgical records)
  - Structured interview
  - Written self-report
  - No description
4. Demonstration that outcome of interest was not present at start of study
  - Yes
  - No

## Comparability

1. Comparability of cohorts on the basis of the design or analysis
  - Study controls for _____________ (select the most important factor)
  - Study controls for any additional factor (This criteria could be modified to indicate specific control for a second important factor.)

## Outcome

1. Assessment of outcome
  - Independent blind assessment
  - Record linkage
  - Self-report
  - No description
2. Was follow-up long enough for outcomes to occur
  - Yes (select an adequate follow-up period for outcome of interest)
  - No
3. Adequacy of follow-up of cohorts
  - Complete follow up—all subjects accounted for
  - Subjects lost to follow-up unlikely to introduce bias—small number lost—> ____ % (select an adequate %) follow-up, or description provided of those lost)
  - Follow-up rate < ____% (select an adequate %) and no description of those lost
  - No statement

**Table 6:** Risk of bias assessment for randomised controlled trials (RCTs): ROB-1

	Sequence generation	Allocation concealment	Blinding: participant and personnel	Blinding: outcome assessors	Incomplete outcome data	Selective reporting	Other bias
Cox et al. [56]	L	L	H	L	H	L	L
Fiuza-Luces et al. [63]	L	L	H	L	L	L	L
Fiuza-Luces et al. [132]	L	L	H	L	H	L	L
Gaser et al. [64]	L	H	?	?	L	L	L
Hamari et al. [65]	?	L	H	L	H	L	L
Hartman et al. [57]	L	L	H	?	H	L	L
Hinds et al. [66]	L	L	H	H	L	L	L
Huang et al. [71]	L	?	?	?	L	L	L
Jacobs et al. [74]	L	?	?	?	H	L	L
Kaushal et al. [58]	?	?	?	?	?	L	L
Khodashenas et al. [59]	?	?	?	?	?	L	L
Kowaluk et al. [67]	?	?	?	?	H	L	L
Lam et al. [68]	L	L	H	H	L	L	L
Li et al. [47]	L	L	?	?	?	L	L
Marchese et al. [60]	?	?	H	L	?	L	L
Masoud et al. [61]	H	H	H	H	L	L	L
Morales et al. [99]	L	L	H	L	H	L	L
Moyer-Mileur et al. [51]	?	?	?	?	L	H	L
Saultier et al. [69]	L	?	?	?	H	L	L
Schechter et al. [72]	L	L	H	L	L	L	L
Senn-Malashonak et al. [73]	?	?	?	?	H	L	L
Sonoda et al. [82]	L	L	H	?	L	L	L
Stössel et al. [70]	L	L	H	H	H	L	L
Waked et al. [62]	L	L	?	?	L	L	L

*H* high, *L* low;?, unclear or not reported
