# Supplementary material for: Nutrition and physical activity interventions delivered to children and young people during cancer treatment: a systematic literature review
Source: Support Care Cancer. 2026 Sep 16;34(10):978. doi: 10.1007/s00520-026-11194-9 (PMC13582132; doi:10.1007/s00520-026-11194-9)
Supplement: Supplementary file 1 [file 520_2026_11194_MOESM1_ESM.docx]

Supplementary material

Supplementary Table S1. Nutrition studies

| Author (year)  Location | Study design  Recruitment period | Sample size  Age (mean ± SD; range) | Sex, n (%) | Ethnicity | Cancer diagnosis n (%) | Type of intervention  Frequency | Timing of intervention  Duration | Follow up | Outcome measures* |
| --- | --- | --- | --- | --- | --- | --- | --- | --- | --- |
| Napartuk 2023 [48] Montreal, QC, Canada | Feasibility cohort study  February 2018 - December 2019 | n=36  7.88 (5.0); 1.57-17.1 | M: 19 (52.8%) F: 17 (47.4%) | NR | Leukaemia 18 (50%),  Lymphoma 9 (25%),  Sarcoma 4 (11.1%),  Other 5 (13.9%). | Individualised nutritional counselling as part of a multidisciplinary program | 1-3 months post diagnosis  12 months | 0, 6 and 12 months | **Anthropometry:** BMI z-score, MUAC, TSFT  **Dietary intake:** 24h dietary recall**/**3-day food record: Diet composition, calorie intake |
| Hill 2018 [44]  Fort Worth, Texas, USA | Retrospective  IG: Treated between January 1, 2013 - December 31, 2015.  Control: Diagnosed January 1, 2011 - December 31, 2012. | Intervention: n=33  <5 years: 20 (60.6%),  >5 years: 13 (39.4%) | M: 20 (60.6%),  F: 13 (39.4%) | White: 17 (51.5%) Hispanic: 14 (42.4%) Other: 2 (6.1%) | ALL: Low/SR 18 (54.5%), HR/VHR 15 (45.5%) | Nutritional counselling with educational resources  3 visits | Maintenance  6 months | Monthly for 12 months of maintenance | **Anthropometry:** BMI z-score |
|  |  | Control: n=34  <5 years 21 (61.8%),  >5 years 13 (38.2%) | M: 18 (52.9%)  F: 16 (47.1%) | White: 24 (70.6%) Hispanic: 8 (23.5%) Other: 2 (5.9%) | ALL: LR/SR 15 (44.1%), HR/VHR 19 (55.9%) | Historical control |  |  |  |
| Li 2016 [47]  Houston, Texas, USA | RCT  NR | Intervention: n=12  7-12 years: 6 (50%), 13-18 years 6 (50%) | M: 9 (75%) F: 3 (25%) | White: 6 (27%) African American: 1 (5%) Hispanic: 13 (59%) Asian: 1 (5%) Mixed race: 1 (5%) | B-cell: SR 4 (33.3%), HR 5 (41.7%), Relapse 1 (8.3%) T-cell 2 (16.7%) | Nutritional counselling  Monthly | Maintenance  12 months | 0, 6 and 12 months | **Anthropometry:** BMI percentile  **Dietary intake:** 3-day 24-hour diet recall: calorie intake, micronutrient intake |
|  |  | Control: n=10  7-12 years: 6 (50%),  13-18 years 4 (40%) | M: 5 (50%) F: 5 (50%) |  | B-cell: SR 3 (30%), HR 6 (60%), T-cell 1 (10%) | Control |  |  |  |
| Moyer-Mileur 2009  [51] Salt Lake City, Utah USA | RCT  NR | Intervention: n=6  7.2 (0.7) | M: 3 (50%) F: 3 (50%) | White: 92% Nothing further reported | SR ALL | Home-based exercise program with dietitian reviews  Monthly | Maintenance  12 months | 0, 3, 6, 9, and 12 months | **Anthropometry:** BMI z score  **Dietary intake:** 3-day food record: energy and nutrient intake  **Physical activity:** Pedometer: steps/day |
|  |  | Control: n=7  5.9 (0.7) | M: 4 (57.1%) F: 3 (42.9%) |  | SR ALL | Control |  |  |  |
| Orgel 2021 [49]  Los Angeles, USA | Non-RCT  IG: May 2016 to March 2019 | Intervention: n=40  15 (3.0) | M: 24 (60%) F: 16 (40%) | Not Hispanic: 8 (20%) Hispanic: 26 (65%) NR: 6 (15%) | HR ALL | Nutritional counselling and exercise program  Weekly | Induction  4 weeks | 0 and 4 weeks | **Anthropometry:** BMI, DXA: FM, LM  **Clinical:** MRD, biomarkers  **Dietary intake:** 3-day food record, 24H recall: %adherence to diet  **Feasibility:** Adherence to the intervention. |
|  | Control: January 2008 to March 2014 | Control: n=80  14.7 (2.5) | M: 43 (54%) F: 37 (46%) | Not Hispanic: 14 (18%) Hispanic: 66 (83%) | HR ALL | Historical control: standard care |  |  |  |
| Viscardi 2021  [45]  Chile | Cohort study  NR | n=12  6.7 years | M: 8 (66.7%) F: 4 (33.3%) | NR | ALL: 8 (66.7%) Hepatic 1 (8.3%) Biliary 1 (8.3%) LCH 1 (8.3%) Astrocytoma 1 (8.3%) | Nutrition education workshops  6 workshops | NR  3 months | 0 and 3 months | **Dietary intake:** 24h dietary recall. 3-day food FFQ: kcal, protein, carbohydrates, fats, vegetables, fruit,  **Feasibility:** Workshop attendance. |
| Walters 2021 [46]  USA | Feasibility cohort study Multicentre.  June 2017 - August 2019 | n=23  9.7 (4.4); 5-19 | M: 12 (52.2%) F: 11 (47.8%) | White: 12 (52.2%) Black or African American: 3 (13%) Adian: 2 (8.7%) Other: 5 (21.7%) Unknown: 1 (4.3%) | ALL: HR 9 (39.1%), IR 3 (13%), LR 11 (47.8%) | Nutrition counselling  0-3 months: weekly  3-6 months: monthly | Induction  6 months | 0, 3 and 6 months | **Anthropometry:** BMI.  **Dietary intake:** 24h diet recall: Calories, carbohydrates, protein, sugar, added sugar, dietary fibre, glycaemic index, vegetables, fruit, grains.  **Feasibility:** Adherence, Acceptability. |
| Zhang 2019 [50] | Feasibility cohort study Multicentre.  2014 -2016 | n=15  6.1 (2.0) | M: 11 (73.3%) F: 4 (26.7%) | Non-Hispanic 9 (60%) Other 6 (40%) | ALL | Self-guided home nutrition and activity program with lifestyle coaching  Weekly | Maintenance or within 2 years of treatment completion  12 weeks | 0 and 6 months | **Anthropometry:** Weight, height, waist circumference  **Dietary intake:** 24hour dietary recall: fruit, vegetables, grains, meat, legumes, dairy, protein, carbohydrates, fat, fibre, sodium, added sugar, healthy eating index, calorie intake.  **Physical activity:** Actigraph: physical activity intensity level minutes/day |

ALL: Acute lymphoblastic leukaemia; CG: control group; F: female; FM: fat mass; HR: high risk; IG: intervention group; IR: intermediate risk; LR: low risk: M: male; NR: not reported; SR: standard risk; VHR: very high risk. LCH: Langerhans cell histiocytosis. BMI: body mass index. FFQ: food frequency questionnaire. MUAC: mid upper arm circumference. TSFT: triceps skin fold thickness. Outcome measures*: reported outcomes relevant to this review

Supplementary Table S2. Nutrition interventions

| Author (year) | Intervention type | Aim | Diagnoses | Timing | Age  Mean (SD) | Control | Provider | Setting | Frequency | Duration | Follow up |
| --- | --- | --- | --- | --- | --- | --- | --- | --- | --- | --- | --- |
| Napartuk 2023 [48] | Nutrition counselling and PA | Prevent short- and long-term metabolic health complications | Mixed | 1-3 months post diagnosis | 8.5 (5.1) years | No control | Dietitian | In hospital and outpatient | 2 monthly | 12 months | Baseline and 12 months (+-2 months) |
| Viscardi 2021 [45] | Group nutrition education workshops | Influence food choices which contribute to the development of healthy eating habits and nutritional status. | Mixed (67% ALL) | Not reported | 6.7 years | No control | NR | NR | 6 workshops | 3 months | 0 and 3 months |
| Orgel 2021 [49] | Nutrition counselling and exercise | >20% caloric deficits during induction, reduce fat intake/ glycaemic load, and increase physical activity. | HR-ALL | Induction | 15 (3.0) years | Historical control | Dietitian, exercise physiologist and physiotherapist | In hospital and outpatient appointment | Weekly | 4 weeks | Baseline and end of induction |
| Walters 2021 [46] | Nutrition counselling | Determine if a dietary intervention is feasible and minimizes weight gain | ALL | Induction or EOI | 9.7 (4.4) years | No control | Registered dietitian and study staff | In hospital, outpatient, and telemedicine | 0-3 months :1x/week  3-6 months: 1x/month | 6 months | 0, 3 and 6 months |
| Zhang 2019 [50] | Nutrition and activity program | Behaviour change: positive parenting style, healthy eating, and increase physical activity. | ALL | Maintenance | 6.1 (2.0) years | No control | Lifestyle coach/self-guided online | Home-based | Weekly | 3 months | Baseline and 6 months |
| Hill 2018 [44] | Nutrition counselling | Evaluate the impact of an early, 3-visit nutrition intervention on weight gain | ALL | Maintenance | 60.6% <5, 39.4% >5years | Historical control | Registered dietitian/ nutritionist | Outpatient | 3 visits | 6 months | Monthly during maintenance. |
| Li 2016 [47] | Nutrition counselling | Assess the initial efficacy of routine nutritional counselling on increasing healthy dietary behaviours, minimizing excess weight gain, and preventing future prevalence of overweight/ obesity | ALL | Maintenance | 7-18 years | RCT | Research dietitian | Outpatient at the time of clinic visits or via phone | Monthly | 12 months | Baseline, 6 months, 12 months |
| Moyer-Mileur 2009 [51] | Exercise and nutrition education program | effect on cardiovascular fitness, strength, and flexibility and feasibility | ALL | Maintenance | 7.2 (0.7) years | RCT | Registered dietitian | Home-based Physical activity | Monthly | 12 months | Baseline, 3m, 6m, 9m, 12m. |

ALL: Acute lymphoblastic leukaemia; EOI: end of induction; RCT: randomised controlled trial; HR: hight risk; NR: not reported

Supplementary Table S3. Physical activity studies

| Author (year) Location | Study design Recruitment | | | Sample size Age (mean ± SD; range) | | Sex, n (%) | | Ethnicity | | Cancer diagnosis  n (%) | | Type of intervention | | | Timing of intervention Duration | | | Follow up | | | | Outcome measures | | | | | |  |
| --- | --- | --- | --- | --- | --- | --- | --- | --- | --- | --- | --- | --- | --- | --- | --- | --- | --- | --- | --- | --- | --- | --- | --- | --- | --- | --- | --- | --- |
| Cox 2018 [56] USA & Canada | Multi centre RCT  NR | | | Intervention: n=53,  4–9 years 19 35.8%,  10–14 years 19 35.8%,  15–18 years 15 28.3% | | M: 34 (64.2%) F: 19 (35.8%) | | White non-Hispanic 31 58.5%, Hispanic 9 17.0%, Black non-Hispanic 3 5.7%, Other 10 18.9% | | ALL | | Home physical activity program | | | Within 10 days of diagnosis  135 weeks | | | T0: baseline t1: 6-7 weeks t2: 8-9 weeks post induction t3: 135 weeks | | | | Physical function: 6MWT, HGS, Knee extension, ankle DF, BOT-2 QOL: HRQOL child health Body composition: BMD Physical activity: Accelerometer, PA logs | | | | | |  |
|  |  | | | Control: n=53,  4-9 years 18 (34.0),  10-14 years 20 (37.7),  15-18 years 15 (28.3) | | M: 36 (66.7%) F: 18 (33.3%) | | White 37 68.5%, Hispanic 7 13.0%, Black non-Hispanic 7 13.0%, Other 3 5.6% | | ALL | | Control | | |  | | |  | | | |  | | | | | |  |
| Eshbenshade 2014 [102]  USA | Prospective cohort study NR | | | n=17, 7.4 (2.0) years | | M: 12 (70.6%) F: 5 (29.4%) | | NR | | ALL | | Home exercise program | | | Maintenance  6 months | | | Baseline,  6 months | | | | Anthropometry: BMI percentile Physical function: 6MWT, Flexibility (sit and reach), ankle DF ROM, knee extension dynamometer strength, BOT-2. | | | | | |  |
| Gohar 2011 [93] USA | Prospective cohort study September 2008 - February 2009 | | | n=9, median 4.0; 2-14 years | | M: 3 (33.3%) F: 6 (66.7%) | | NR | | ALL: SR 7, T-cell 1, HR 1 | | In-hospital physical therapy- and home exercise program | | | 0-2 weeks from diagnosis 6-7 months | | | End of induction, consolidation, IM and DI | | | | Physical function: Gross motor function QOL: PedsQL 4.0 generic core scale | | | | | |  |
| Hartman 2009 [57] the Netherlands | RCT April 2001 -September 2004 | | | Intervention: n=25, median 5.3; 1.3-15.6  Control: n=26, median 6.2; 1.7-17.1 | | M: 14 (56%) F: 11 (44%)  M: 16 (62%)  F: 10 (38%) | | NR  NR | | ALL  ALL | | Exercise program | | | Diagnosis 2 years | | | Diagnosis, 2 years, 1 year post EOT | | | | Anthropometry: Weight, height, BMI Body composition : LM, BF%, BMD Physical function: Ankle dorsiflexion, Movement ABS (>4 years), BSID-II (<3.5 years) | | | | | |  |
| Hooke 2016 [105] USA | Multicentre Prospective cohort study NR | | | n=16, 8.69 (3.09) | | M: 5 (31%) F: 11 (69%) | | Caucasian non-Hispanic 15 (94%) Caucasian Hispanic 1 (6%) | | ALL: SR 12 (75%), HR 4 (25%) | | Physical activity (fitness tracker) | | | Maintenance  3 weeks | | | Baseline (day -17 to -15) Week -2 (days -14 to -8) Week -1 (day -7 to -1) Steroid pulse (day 1 to 5) | | | | Physical Activity: Steps/day Fatigue: Fatigue scale | | | | | |  |
| Kaushal 2013 [58] India | RCT | | | Total cohort: n=36 | | Total cohort:  M: 31 (86.1%) F: 5 (13.9%) | | NR | | ALL | | Aerobic exercise | | | Start of treatment  3 weeks | | | Pre and post (3 weeks) | | | | Fatigue: Piper scale | | | | | |  |
|  |  |  |  |  |  |  |  |  |  |  |  | Control: Relaxation | | |  |  |  |  |  |  |  |  |  |  |  |  |  |  |
| Khodashenas 2017 [59] Iran | RCT 2015-2016 | | | Intervention: n=10, 10.1;5-12  Control: n=10, 8.8; 5-12 | | M: 12 F: 8 | | NR  NR | | ALL  ALL | | Aerobic exercise program  Standard care | | | Post induction to <1 year after EOT 12 weeks | | | Baseline and 12 weeks | | | | QOL: PedsQL 4.0 translated into Persian | | | | | |  |
| Marchese 2004 [60] Philadelphia, USA | RCT  NR | | | Intervention: n=13, 7.6; 4.3-10.6 | | M: 8 (61.5%) F: 5 (38.5%) | | NR | | ALL: SR: 10 (76.9%), HR: 3 (23.1%) | | Physical therapy | | | Maintenance  4 months | | | Pre and post (4 months) | | | | Physical function: Knee extension dynamometer strength, ankle DF ROM, TUDS, 9MWR  QOL: PedsQL 3.0 | | | | | |  |
|  |  |  |  | Control: n=15, 8.6; 5.1-15.8 | | M: 12 (80%) F: 3 (20%) | | NR | | ALL: LR: 2 (13.3%), SR: 9 (60%), HR: 4 (26.7%) | | Control | | |  | | |  | | | |  |  |  |  |  |  |  |
| Masoud 2023  [61]  Saudi Arabia | RCT | | | Intervention: n=22, 9.04 (2.29) | | M: 12 (54.5%) F: 10 (45.5%) | | NR | | HR ALL 17 (37.80%), SR ALL 5 (11.10%) | | Exergaming | | | Any phase  3 weeks | | | T1: baseline  T2: Post 1 week  T3: Post 3-week intervention | | | | Physical function : 6MWT  Physical activity:  Godin-Shepard leisure time PA questionnaire  Fatigue : PedsQL MFS | | | | | |  |
|  |  |  |  | Control: n=23, 9.04 (2.47) | | M: 13 (56.5%) F: 10 (43.5%) | | NR | | ALL: HR 15 (33.30%), SR 8 (17.80%) | | Control: Education session | | |  | | |  |  |  |  |  |  |  |  |  |  |  |
| Moyer-Mileur 2009  [51] Salt Lake City, Utah USA | RCT  NR | | | Intervention: n=6  7.2 (0.7) | | M: 3 (50%) F: 3 (50%) | | White: 92% Nothing further reported | | SR ALL | | Home-based exercise program with dietitian reviews  Monthly | | | Maintenance  12 months | | | 0, 3, 6, 9, and 12 months | | | | Anthropometry: BMI z score  Dietary intake: 3-day food record: energy and nutrient intake  Physical activity: Pedometer: steps/day | | | | | |  |
|  |  | | | Control: n=7  5.9 (0.7) | | M: 4 (57.1%) F: 3 (42.9%) | |  | | SR ALL | | Control | | |  | | |  | | | |  |  |  |  |  |  |  |
| Orgel 2021 [49]  Los Angeles, USA | Non-RCT  IG: May 2016 to March 2019 | | | Intervention: n=40  15 (3.0) | | M: 24 (60%) F: 16 (40%) | | Not Hispanic: 8 (20%) Hispanic: 26 (65%) NR: 6 (15%) | | HR ALL | | Nutritional counselling and exercise program  Weekly | | | Induction  4 weeks | | | 0 and 4 weeks | | | | Anthropometry: BMI, DXA: FM, LM  Clinical: MRD, biomarkers  Dietary intake: 3-day food record, 24H recall: %adherence to diet  Feasibility: Adherence to the intervention. | | | | | |  |
|  | Control: January 2008 to March 2014 | | | Control: n=80  14.7 (2.5) | | M: 43 (54%) F: 37 (46%) | | Not Hispanic: 14 (18%) Hispanic: 66 (83%) | | HR ALL | | Historical control: standard care | | |  | | |  | | | |  |  |  |  |  |  |  |
| San Juan 2007 [91] | Prospective cohort study | | | n=7, 5.1 (1.2) | |  | |  | | ALL | | Exercise program | | | Maintenance  8 weeks | | | Baseline Post training (8 weeks) | | | | Physical function: Strength (6RM: Seated bench row, seated row, seated leg press), TUDS, TUG (3M, 10M) | | | | | |  |
| San Juan 2007 [97] Madrid, Spain | Prospective cohort study | | | n=7, 5.1 (1.2) | | M: 4 (57.1%) F: 3 (42.9%) | | NR | | ALL | | Exercise program | | | Maintenance 16 weeks | | | Baseline  Post training (16weeks)  Detraining (+20 weeks) | | | | Physical function: Strength (5RM seated bench row, seated row, seated leg press), TUDS, TUG, passive and active dorsiflexion ROM, VO2 peak.  QOL: CHIP-CE/CRF | | | | | |  |
| Tanner 2017 [100] Minnesota, USA | Program evaluation N/A | | | n=135, 5.79; 1-22 | | M: 56 (41%) F: 79 (59%) | | Caucasian 94 (70%), Black/African American 8 (6%), Hispanic 8 (6%), Asian 15 (11%), Other/mixed 6 (4%), Unknown 4 (3%). | | SR ALL 88 (65%), HR ALL 47 (35%) | | Physical therapy | | | 6-8 weeks post diagnosis NR | | | Baseline, end of IM, end of DI, maintenance cycle 1, 3 and 6. | | | | Physical function: Ankle ROM, floor to stand, gait, balance, PDMSII or BOT-2 Physical activity: Lansky play performance scale, Karnofsky | | | | | |  |
| Waked 2018 [62] Egypt | RCT NR | | | Intervention: n=23, 9.26 (2.39) | | M: 15 (34.8%) F: 8 (65.2%) | | NR | | ALL | | Exercise program | | | Maintenance 12 months | | | Baseline, 6 months, 12 months | | | | Body composition: BMD, lean body mass, BF% | | | | | |  |
|  |  |  |  | Control: n=23, 9.91 (2.09) | | M: 18 (78.3%) F: 5 (21.7%) | | NR | |  | |  | | |  | | |  |  |  |  |  |  |  |  |  |  |  |
| Wright 2003 [86] Canada | Prospective cohort study NR | | | Intervention: n=40 5.5 years | | M: 50% F: 50% | | NR | | ALL: HR: 37.5% | | Physical therapy education for ankle ROM | | | Not specified 2 years | | | Not specified | | | | Physical function: Ankle DF (active and passive) | | | | | |  |
|  |  | | | Control: n=54  4.8 years | | M: 64.8% F: 35.2% | | NR | | ALL  HR: 46.3% | | Historical control, >12 months post treatment completion | | |  | | |  | | | |  | | | | | |  |
| Yeh 2011 [75]  Taiwan | Prospective cohort study June 2006 - July 2008 | | | Intervention: n=12, 11.01 (3.56) | | M: 6 (50%) F: 6 (50%) | | NR | | ALL | | Home-based aerobic program | | | Maintenance 6 weeks | | | Baseline, weekly for 1-6 weeks. 1 month post intervention. | | | | Physical activity: Physical activity level Fatigue: PedsQL MFS | | | | | |  |
|  |  |  |  | Control: n=10, 12.48 (3.86) | | M: 6 (60%) F: 4 (40%) | | NR | |  | | Control - routine care | | |  | | |  |  |  |  |  | | | | | |  |
| Zhang 2019 [50] | | Feasibility cohort study Multicentre.  2014 -2016 | | n=15  6.1 (2.0) | | M: 11 (73.3%) F: 4 (26.7%) | | Non-Hispanic 9 (60%) Other 6 (40%) | | ALL | | | Self-guided home nutrition and activity program with lifestyle coaching  Weekly | | | Maintenance or within 2 years of treatment completion  12 weeks | | | 0 and 6 months | | | | | Anthropometry: Weight, height, waist circumference  Dietary intake: 24hour dietary recall: fruit, vegetables, grains, meat, legumes, dairy, protein, carbohydrates, fat, fibre, sodium, added sugar, healthy eating index, calorie intake.  Physical activity: Actigraph: physical activity intensity level minutes/day | | |  |  |
| Atkinson 2023 [101] Adelaide, Australia | Prospective cohort study May 2019 - June 2021 | | | n=23  15.7 (4.9) | | M: 9 (43%) F: 12 (57%) | | NR | | Sarcoma 19% Leukaemia 14% Lymphoma 19%  CNS Germ Cell Tumour 1 4% MBL 5 23% Pilocytic Astrocytoma 3 14% Sacral meningioma-papillary variant, G3 1 4% Myxopapillary Ependymoma 1 4%. | | Robotic rehabilitation using an advanced robotic gait training device | | | During treatment or recently completed  6 weeks | | | Baseline and 6 weeks | | | | Physical function: 6MWT, VO2 peak, Sit and reach, back and leg strength,  Physical activity: PA level, questionnaire Fatigue: FACIT fatigue QOL: QOL score | | | | | |  |
| Bluth 2016 [135] USA | Prospective cohort study NR | | | n=64 11.2 (3.9) years | | M: 43 (67.2%) F: 21 (32.8%) | | Caucasian 31%, African American 28%, Hispanic 33%, Asian 5% | | NR | | Martial arts | | | NR 1 hour | | | Pre and post sessions | | | | Other: Pain scale | | | | | |  |
| Caru 2020  [106]  Montreal, Canada | Prospective cohort study December 2017 - July 2019 | | | n=16  12.1 (3.6) | | M: 8 (50%)  F: 8 (50%) | | NR | | ALL 4 (25.0)  EWS 3 (18.8) Burkitt lymphoma 3 (18.8)  HL 2 (12.5) Osteosarcoma 2 (12.5) RMS 1 (6.3)  Wilms tumour 1 (6.3) | | Home based physical activity program | | | 2-4 weeks post diagnosis | | | Baseline and 6 weeks | | | | Physical activity : MVPA minutes/day | | | | | |  |
| Corr 2017 [87], USA | Prospective cohort study 2012-2016 | | | Intervention: n=14, 13.5 (3.5) | | M: 10 (71.4%) F: 4 (28.6%) | | Race: Black 1 (7.1) Other 4 (28.6) White 9 (64.3) | | Osteosarcoma 11 (78.6) EWS 2 (14.3) Chondroblastoma 1 (7.1) | | Prehabilitation | | | Diagnosis 10-12 weeks (until local control surgery) | | | Baseline (dx), pre surgery (10-12wks), post-surgery (20-22wks) | | | | Feasibility  Physical function: The Functional Mobility Assessment ROM: actively and passively using a goniometer, hip flexion and extension, hip abduction and adduction, hip internal and external rotation, knee flexion and extension, and ankle DF and plantar flexion. TUG, TUDS, 9MWR, Strength: HGS, Hip flexion, knee extension, and ankle DF | | | | | |  |
|  | 2008-2012 | | | Control: n=35, 13.1 (3.5) | | M: 19 (54.3%) F: 16 (45.7%) | | Black 10 (28.6) Other 5 (14.3) White 20 (57.1) | | Osteosarcoma 35 (100%) | | Historical control | | | | | |  |  |  |  |  |  |  |  |  |  |  |
| Dalla Santa 2023 [103], Australia | Prospective cohort study September 2019 - April 2020 | | | n=14, 8; 4.91-14.99) | | M: 10 (71%) F: 4 (29%) | | NR | | ALL 7 (50%) Nephroblastoma 1 (7.14%) EWS 1 (7.14%) non-HL 2 (14.30%) ALCL 1 (7.14%) HL 2 (14.30%) | | Physiotherapy | | | Diagnosed <3m ago 8 weeks | | | Pre and post | | | | Physical function: 6MWT, Lansky play performance scale. Neuropathy Score (TNS©-PV). Ankle DF passive ROM with knee flexion and knee extension. Floor rise (CMAS). MABC-2. QOL: PedsQL 4.0 Feasibility and acceptability | | | | | |  |
| Fukuhara 2020 [139] USA | Prospective cohort study July 2016-october 2016 | | | n=15, 11.8; 7-17 | | M: 8 (53.3%) F: 7 (46.7%) | | Black 1 (6.7), Caucasian 6 (40.0), Hispanic 8 (53.3) | | ALL 10 (66.7%), NBL 2 (13.3%), AML 1 (6.7%), Burkitt 1 (6.7%), Wilms 1 (6.7%) | | Yoga session | | | During treatment 1 session | | | Pre and post session | | | | Anxiety : Spielberger state trait anxiety scale Pain: Wong baker faces pain scale | | | | | |  |
| Fuiza-Luces 2017 [63] Spain  NCT01645436 | RCT September 2012-March 2015 | | | Intervention: n=24, 10 (1); 4-16 | | M: 17 (71%) F: 7 (29%) | | NR | | HL 4%. Burkitt's lymphoma 21%,  T-LLy 13%,  RMS 8%, non-RMS 4%. EWS 25%, Osteosarcoma 13%. NBL 4%, Germinomas 4%, Non germinomas 4%. | | Exercise intervention | | | From diagnosis ~19 weeks (duration of neoadjuvant treatment) | | | treatment initiation, end of treatment (~20 weeks), 2 months (8 weeks) post end of treatment. | | | | Physical function: 5RM of bench press, leg press, and lateral row. VO2 peak. Timed up and go (3m), timed up and down stairs.  Physical activity: PA level (accelerometery) Quality of life: PedsQL 3.0 cancer module.  Anthropometry: Weight and BMI | | | | | |  |
|  |  | | | Control: n=25, 11 (1); 5-17 | | M: 18 (72%) F: 7 (28%) | |  | |  | | Control | | |  | | |  | | | |  | | | | | |  |
| Fiuza-Luces 2017 [132]  NCT01645436 | As above | | | Intervention: n=9, 11 (4) years | | M: 7 (77.8%)  F: 2 (22.2%) | |  | | Synovial sarcoma 1, EWS 4, osteosarcoma 2, Burkitt lymphoma 1, neuroblastoma 1. | |  | | | 17 (5) weeks (duration of neoadjuvant treatment) | | | Baseline, post treatment, detraining | | | | Clinical: Immune cell function  Adherence.  Physical activity: MVPA (accelerometer) | | | | | |  |
|  |  | | | Control: N=11  12 (4) years | | M: 6 (54.5%)  F: 5 (45.5%) | |  | | Synovial sarcoma 2, EWS 3, Osteosarcoma 1, HL 2, DLBCL 1, T cell LLy 1, ganglioneuroblastoma 1. | |  | | | 15 (4) weeks (duration of neoadjuvant treatment) | | |  | | | |  | | | | | |  |
| Morales 2018 [99]  NCT01645436 | As above | | | N=24, 10 (4); 4-16 | | M: 17 (71%)  F: 7 (29%) | |  | | HL 4% non-HL 34% RMS 8% Synovial sarcoma 4% EWS 25% Osteosarcoma 13% NBL 4% Germinomas 4% Non-germinomas (embryonic carcinoma) 4% | |  | | |  | | | Baseline  Post (mean 19 weeks) | | | |  | | | | | |  |
| Morales 2020   [76]  Spain | Quasi controlled study  January 2013 to June  2018 | | | Intervention: n=68, 11 (3) | | M: 40 (60%)  F: 28 (40%) | | Spanish Caucasian 93%, Hispanic 3%, Other 4% | | ALL: 37% AML: 5%  HL: 6% Non‐HL: 8% Ependymomas and choroid plexus tumour 1% Astrocytoma 1% Intracranial and intraspinal embryonal tumours 3% NBL 6% Renal tumours Osteosarcomas 9% EWS and bone sarcoma 14% RMS 6% Other specified soft tissue sarcomas 1% Intracranial and intraspinal germ cell tumours 3% | | Exercise intervention | | | Start of treatment  Neoadjuvant/ intensive treatment | | | Baseline (diagnosis), end of neoadjuvant treatment (solids) or intensive treatment (leukaemia), 1,2,3,4,5 years post diagnosis | | | | Clinical : Mortality, relapse, length of hospitalisation, economic cost, cardiac function  Anthropometry : BMI | | | | | |  |
|  |  | | | Control: n=101, 11 (4) | | M: 63 (62%)  F: 38 (28%) | | Spanish Caucasian 80%, Hispanic 11%, Other 9% | | ALL 27% AML 8% HL 8% Non‐HL 11% Ependymomas and choroid plexus tumour 2% Intracranial and intraspinal embryonal tumours 4% Other gliomas 2% NBL and ganglioneuroblastoma 4% Renal tumours Nephroblastoma and other nonepithelial renal tumours 1% Osteosarcomas 10% EWS and related sarcomas of bone 11% RMS 3% Other specified soft tissue sarcomas 5% Intracranial and intraspinal germ cell tumours 3% Malignant gonadal germ cell tumours 1% | |  | | |  | | |  | | | |  | | | | | |  |
| Gaser 2022 [64] Germany | RCT  September 2017 - June 2020 | | | Intervention: n=21, 10.2 (4.2) | | M: 15 (71% F: 6 (29%) | | NR | | ALL 11 (52%), AML 1 (5%), NHL 9 (43%), | | Strength training + Standard care exercise program | | | Diagnosis Intensive treatment (~7 months) | | | V0: baseline  V1: after induction  V2: End of consolidation  V3: end of intensive treatment | | | | Physical function: Moon-test Activity scale for kids’ performance (ASKP)  Physical activity: MVPA , step count (accelerometer) Adherence | | | | | |  |
|  |  |  |  | Control: n=20, 9.7 (3.9) | | M: 12 (60%) F: 8 (40%) | | NR | | ALL 14 (70%), AML 3 (15%), NHL 3 (15), Second primary cancer 1 (5%) | | Standard care exercise program | | |  |  |  |  |  |  |  |  |  |  |  |  |  |  |
| Gotte 2018 [77]  Germany | Quasi experimental study | | | Intervention: n=21, at t2: 14.5 (3.9) | | M: 13 (62%)  F: 8 (38%) | | NR | | ALL 9 (43%) AML 1 (5%) Lymphoma 6 (29%) EWS 3 (14%) OSS 1 (5%) Other solid tumour 1 (5%) | | Home based individual exercise program | | | During intensive treatment  6-8 weeks | | | T1: Baseline  T2: After treatment cessation | | | | Physical function : Moon test  QOL:  KINDL questionnaire.  Physical activity  Steps/day (Actigraph), activity level | | | | | |  |
|  |  |  |  | Control: n=19, 15.4 (3.7) | | M: 9 (47%) F: 10 (53%) | | NR | | ALL 4 (21%) Lymphoma 5 (26%) EWS 4 (21%) OSS 2 (11%) Other solid tumour 4 (21%) | | Non-RCT | | |  |  |  | T2: after treatment cessation | | | | | | |  | | |  |
| Govardhan 2019 [107] India | Prospective cohort study | | | n=18, 9.8 years | | M: 9 (50%) F: 9 (50%) | | NR | | Anaplastic ependymoma 6, Medulloblastoma 5, Atypical neurocytoma 1, Glioma 4, CNS Germ cell tumour 1, Retinoblastoma 1 | | Yoga | | | During treatment 4 weeks | | | Baseline 4 weeks | | | | Symptoms | | | | | |  |
| Grimshaw 2024  [98] Australia | Prospective cohort study  2 November 2020-16 June 2021 | | | n=20, 13 (9-11) | | M: 11 (55%)  F: 9 (45%) | | NR | | AML 8 (40%) ALL 7 (35%) Burkitt Lymphoma 2 (10%) HL 1 (5%) NBL 1 (5%) Osteosarcoma 1 (5%) | | Physical activity | | | 4 weeks to 6 months post diagnosis 10 weeks | | | Baseline, post intervention | | | | Physical function:  Movement ABC-2, TUG, TUDS, 6MWT, Timed Rise from the Floor, and 30 second Chair Stand  Physical activity:  Fitbit  QOL: PedsQL Core and Cancer Module | | | | | |  |
| Hamari 2019 [65] Finland | RCT NR | | | Intervention: n= 17, 7.8; 3-16 | | M: 12 (70.5%) F: 5 (29.5%) | | NR | | ALL (SR:IR:HR) 7 (4:2:1), Wilm's Tumour 2, Burkitt lymphoma 1, non-HL 3, HL 1, Other 3 | | Active video game | | | Within 1 week of diagnosis 8 weeks | | | Baseline, 2 months, 6 months, 12 months, 30 months | | | | Physical activity: Activity level Fatigue: PedsQL fatigue Physical function: Movement-ABC-2 Feasibility and acceptability | | | | | |  |
|  |  | | | Control: n=19, 7.9; 3-15 | | M: 14 (73.7%) F: 5 (26.3%) | | NR | | ALL: SR 4, IR 4, HR 2, Burkitt lymphoma 2, non-HL 2, HL 2, Other 3 | | Control | | |  | | |  | | | |  | | | | | |  |
| Hayati 2019 [78] Indonesia | Quasi experimental study NR | | | Intervention: n=32, 11.69 (2.58) | | M: 18 (56.2%) F: 14 (43.8%) | | NR | | Blood cancer: 18 (40.6%). Solid cancer 13 (59.4%) | | Aerobic exercise and relaxation | | | During treatment 5 days | | | Day 1  Day 5 | | | | Fatigue: Childhood fatigue scale Other: Sleep disturbance scale | | | | | |  |
|  |  |  |  | Control: n=32, 12.97 (3.20) | | M: 18 (56.2%)  F: 14 (43.8%) | | NR | | Blood cancer: 43.8 (18). Solid cancer: 56.2 (14) | | Control - standard care | | |  | | |  | | | |  | | | | | |  |
| Hinds 2007 [66] USA | Prospective multi-site RCT NR | | | Intervention: n=14, 13.08 (2.55) | | M: 9 (64%) F: 5 (36%) | | Hispanic/Latino 2 (14%). White 9 (64%). Black 3 (21%) | | Solid tumour 13 (93%). AML 1 (7%) | | Enhanced physical activity | | | During treatment 2-4 days | | | Day 0, 1 , 2, 3 | | | | Fatigue: Fatigue scale Other: Sleep duration and efficiency | | | | | |  |
|  |  |  |  | Control: n=15, 11.92 (3.24) | | M: 3 (20%) F: 12 (80%) | | Hispanic/Latino 2 (13%). White 12 (80%). Black 1 (7%) | | Solid tumour 12 (80%). AML 3 (20%) | | Control | | |  | | |  | | | |  | | | | | |  |
| Hooke 2019 [88] | Prospective cohort study, multicentre  NR | | | Intervention: n=30, 12.0 (3.6) | | M: 19 (63%) F: 11 (37%) | | Caucasian 25 (83%), Hispanic Latino 3 (10%), Black 1 (3%), Native American 1 (3%) | | ALL 7 (22%), Lymphoma 12 (38%), Solid tumour 12 (38%), CML 1 (3%) | | Physical activity coaching | | | >2 months from diagnosis 4 months | | | Every 2 months | | | | Physical activity: MVPA minutes/day Other: Fatigue | | | | | |  |
|  |  | | | Control: n=27, 12.8 (3.3) | | M: 14 (52%) F: 13 (48%) | | Caucasian 21 (78%), Hispanic Latino 1 (4%), Black 2 (7%), Asian 1 (4%), Mixed 2 (7%) | | ALL 12 (4%), Lymphoma 5 (19%), Solid tumour 10 (37%) | | Historical control | | |  | | |  | | | |  | | | | | |  |
| Kawaluk 2022 [67] Poland | RCT January 2019 - January 2020 | | | Intervention: n=10, 11.3 (2.0) | | M: 5 (50%) F: 5 (50%) | | NR | | ALL n = 8  AML n = 2 | | Interactive video games physical activity | | | <6 months from diagnosis 4 weeks | | | Baseline 4 weeks  14 months | | | | Cardiorespiratory fitness: Peak oxygen uptake, peak heart rate, volume of O2 uptake, volume of exhaled CO2, minute ventilation, ventilatory equivalent of carbon dioxide, respiratory quotient, metabolic equivalent of task. Physical activity: Health Behaviour Based on the School-Aged Children questionnaire Other: Children’s effort rating table, energy expenditure (Kaytel equation) | | | | | |  |
|  |  |  |  | Control: n=11, 10.08 (1.9) | | M: 7 (63.6%) F: 4 (36.4%) | | NR | | r ALL (n = 5) and AML (n = 6). | | control | | |  | | |  | | | |  |  |  |  |  |  |  |
| Lam 2018 [68]  China | RCT  October 2015 - October 2016 | | | Intervention: n=37, 12.8 (2.5) | | M: 20 (54.1%) F: 17 (45.9%) | | NR | | Leukaemia 16 (43.2), Lymphoma 7 (18.9), Brain tumour 6 (16.2), Germ-cell and gonadal tumours 4 (10.8), Bone tumour 2 (5.4), Others 2 (5.4) | | Home training program | | | During treatment  9 months | | | Baseline, 6 months, 9 months  PA levels not at 6 months | | | | Fatigue  Fatigue Scale (Chinese version)  Physical activity  The Chinese University of Hong Kong Physical Activity Rating for Children and Youth, Physical Activity Self-Efficacy scale  Physical function  HGS (L+R)  QOL : PedsQL 3.0 cancer module | | | | | |  |
|  |  | | | Control: n=33, 12.5 (2.5) | | M: 15 (45.5%) F: 18 (54.5%) | | NR | | Leukaemia 14 (42.4), Lymphoma 8 (24.2), Brain tumour 5 (15.2), Germ-cell and gonadal tumours 2 (6.1), Bone tumour 2 (6.1), Others 2 (6.1) | | Control | | |  |  |  |  |  |  |  |  |  |  |  |  |  |  |
| Lam 2020 [141] China | Qualitative study July to August 2017, | | | n=23, 12.4 (2.5) | | M: 13 (56.5%) F: 10 (43.5%) | | NR | | Leukaemia 11 (47.8) Lymphoma 5 (21.7) Brain and spinal tumour 4 (17.4) Germ-cell and gonadal tumours 2 (8.7) Bone tumour 1 (4.4) | | Previous participants of home-training program | | | | |  | | | |  | | | | |  | |  |
| Muller 2014 [79] Germany | Prospective cohort study July 2008-July 2010 | | | Intervention: n=10, 15.2 (2.0) | | M: 4 (40%) F: 6 (60%) | | NR | | Osteosarcoma 7  EWS 3 | | Exercise intervention | | | Start of treatment Duration of treatment | | | Baseline, post local control, treatment completion (6 months later), 6 months post treatment completion | | | | BMD: DXA: Lumbar spine, femur, calcaneus Physical activity: MPA (mins), gait cycles | | | | | |  |
|  |  | | | Control: n=11, 12.2 (2.6) | | M: 5 (45.5%) F: 6 (54.5%) | | NR | | Osteosarcoma 7,  EWS 4 | | Non-RCT, separate institution | | |  |  |  |  |  |  |  |  |  |  |  |  |  |  |
| Nielson 2018 [96] Denmark | Prospective cohort study January 2013 and April 2016 | | | n=75, 11.3 (3.1) | | M: 46 (61%) F: 29 (39%) | | NR | | Leukaemia 31 (41%), Lymphoma 14 (19%), Extracranial solid tumours 21 (28%), CNS tumours 9 (12%) | | Physical activity and social support | | | Diagnosis | | | Baseline  3 months | | | | Physical function: Cardiopulmonary exercise test (CPET); TUG; sit-to-stand; flamingo balance; and HGS Other: Barriers to physical function testing | | | | | |  |
| Nielsen 2020 [80] Denmark | Prospective cohort study January 2013 - February 2018 | | | Intervention: n=120, 11.2 (3.1) | | M: 75 (62.5%) F: 45 (37.5%) | | NR | | Leukaemia 47 (39%) Lymphoma 22 (18%) Extracranial solid tumours 35 (29%) CNS tumour 11 (9%) Other haematological disease 5 (4%) | | Physical activity and social support | | | Diagnosis | | | Baseline  3 months  6 months | | | | Physical function: Cardiopulmonary exercise test (CPET); timed-up-and-go; sit-to-stand; flamingo balance; and HGS Other: Barriers to physical function testing | | | | | |  |
|  |  |  |  | Control: n=50, 11.0 (3.2) | | M: 31 (57%) F: 23 (43%) | | NR | | Leukaemia 23 (46%) Lymphoma 8 (16%) Extracranial solid tumours 14 (28%) CNS tumour 5 (10%) | |  | | |  |  |  |  |  |  |  |  |  |  |  |  |  |  |
| Fridh 2023 [104] Denmark | Prospective cohort study January 2013 - February 2018 | | | Intervention: n=75, 13.4 (3.1) | | M: 45 (61%) F: 30 (39%) | | NR | | Leukaemia 37 (49%) Lymphoma 16 (21%) Extracranial solid tumours 16 (21%) CNS tumour 4 (5%) Other haematological disease 3 (4%) | | Physical activity and social support | | |  | | | 1 year post treatment completion | | | | Cardiopulmonary fitness: VO2peak measured with the cardiopulmonary exercise test (CPET) modified Godfrey protocol on an electronically braked cycle ergometer  Physical function: Sit-to-Stand, TUG, HGS. | | | | | |  |
|  | Control group - Other centre | | | Control: n=33, 13.5 (2.5) | | M: 19 (58%)  F: 14 (42%) | | NR | | Leukaemia 16 (48%) Lymphoma 7 (21%) Extracranial solid tumours 9 (27%) CNS tumour 1 (3%) | |  | | |  | | |  | | | |  | | | | | |  |
| Peterson 2022   [131]  Denmark' | Qualitative descriptive study  September 2019 - May 2020 | | | n=18, 13.5; 11-18 | | M: 10 (55.6%)  F: 8 (44.4%) | | NR | | Leukaemia 11, CNS tumour 1, Extracranial solid tumours 3, HL 1, Non-HL 2 | |  | | |  | | | Mean 2.5 years post EOT | | | |  | | | | | |  |
| Orsey 2017 [109]  USA | Prospective cohort study  NR | | | n=10, 8-13 years: 30%  13-18 years 70% | | M: 2 (80%)  F: 8 (80%) | | African American: 1 (10%), Hispanic 1 (10%), White 8 (80%) | | ALL 2 (20%), B-cell lymphoma 1 (10%), germ cell tumour 1 (10%), HL 2 (20%), MBL 1 (10%), nerve sheath tumour 1 (10%), non-HL 1 (10%), osteosarcoma 1 (10%). | | Yoga | | | During treatment  8 weeks | | | Baseline  Week 8 | | | | Fatigue:  Fatigue scale.  Physical activity : Actigraph  QOL:  PedsQL 4.0, PedsQL cancer 3.0. | | | | | |  |
| Oscwald 2023 [136] Germany | Prospective cohort study May 2015-April 2017 | | | n=11, 7-17years | | M: 8 (72.7%) F: 3 (27.3%) | | NR | | Germ cell tumour 2, EWS 1, Lymphoma 4, Leukaemia 3, RMS 1 | | Whole-body vibration training and exercise program | | | NR Variable (range 7-52 weeks) | | | n/a | | | | Feasibility | | | | | |  |
| Ouyang 2019 [81] China | Prospective cohort study NR | | | Intervention: n=57,  4-6y: 26.3%, 7-11y: 49.1%, 12-14y: 17.6%, 15-18y: 7.0% | | M: 40 (70.2%) F: 17 (29.8%) | | NR | | Leukaemia 10 (17.5), Lymphoma 20 (35.1), Solid tumour 27 (47.4) | | Group physical activity | | | During treatment 3 months (12 sessions) | | | T0: baseline, week 3, week 6, week 9, week 12 | | | | Physical activity: MVPA, light PA (Children’s Leisure Time Activities Study Survey-Chinese) Fatigue: MSAS | | | | | |  |
|  | Control group - Other centre | | | Control: N=57, 4-6 y: 31.6%, 7-11y: 42.1%, 12-14y: 19.3%, 15-18y: 7.0% | | M: 40 (70.2%) F: 17 (29.8%) | | NR | | Leukaemia 12 (21.1), Lymphoma 20 (35.1), Solid tumour 25 (43.8) | | Standard care | | |  | | |  | | | |  | | | | | |  |
| Platschek 2017  [90] | Prospective pilot feasibility cohort study | | | n=9, 11.33 (2.24) | | M: 3 (33.3%  ) F: 6 (66.7%) | | NR | | Leukaemia n = 3, lymphoma n = 3, sarcoma n = 2, NBL n = 1 | | Active video game | | | During treatment 12 weeks | | | Baseline  4 weeks  8 weeks  12 weeks | | | | Mood : Moodmeter  Fatigue : PedsQL MFS | | | | | |  |
| Pouplier 2024 [142] Denmark | Qualitative study on an RCT  April 2021 to April 2022 | | | n=15, 1-5y | | M: 8 (53.3%) F: 7 (46.7%) | | NR | | NR | | Active play | | | From diagnosis 6 months | | | NR | | | | Qualitative observations | | | | | |  |
| Saultier 2021 [69] France | RCT November 2014 and January 2018 | | | Intervention: n=41, 11.4 (0.6) | | M: 23 (56%) F: 18 (44%) | | NR | | Leukaemia 16 (39%) Lymphoma 8 (20%) CNS 7 (17%) Bone 5 (12%) Other tumour 5 (12%) | | physical activity program | | | mean 11.8 (2.0) months from diagnosis  6 months | | | T0: baseline 6 months 12 months | | | | Physical function: 6MWT, HR, Sit and reach, flamingo balance, upper limb strength (medicine ball launch), lower limb strength (Myotest, chair test), trunk muscle endurance (bridge), abdominal muscle endurance (sit-ups) Anthropometry: BMI Body composition : Fat mass, lean mass Quality of life: VSP-A (parent scale), PSI-VSF scale | | | | | |  |
|  |  |  |  | Control: n=39, 11.2 (0.6) | | M: 23 (59%) F: 16 (41%) | | NR | | Leukaemia 15 (38%) Lymphoma 8 (21%) CNS 5 (13%) Bone 7 (18%) Other tumour 4 (10%) | | Control | | |  |  |  |  |  |  |  |  |  |  |  |  |  |  |
| Senoda 2024 [82] Japan | Quasi RCT August 1, 2016, to April 30, 2020 | | | Intervention: n=7, 9.1 (2.2) | | M: 4 (57.1%) F: 3 (42.9%) | | NR | | Leukaemia and lymphoma | | Early physical therapy | | | At admission 40 weeks | | | At admission,  Week 8.  week 40. | | | | Physical function: 6MWT ( 30m)  Quality of life: PedsQL (Japanese version) | | | | | |  |
|  |  |  |  | Control: n=6, 9.7 (2.7) | | M: 5 (83.3%) F: 1 (16.7%) | | NR | |  | | Control - delayed physical therapy (week 8) | | |  | | |  | | | |  | | | | | |  |
| Stein 2019 [108]  Canada | Prospective cohort study March and November 2017 | | | n=10, median 15.1; 11-18 | | M: 3 (30%)  F: 7 (70%) | | NR | | Solid tumour 5 (50) Brain tumour 4 (40) Leukaemia or lymphoma 1 (10) | | Yoga during HSCT | | | During treatment  10 weeks | | | Baseline  5 weeks  10 weeks | | | | QOL:  PedsQL cancer module  Fatigue:  PedsQL MFS  Other  PROMIS | | | | | |  |
| Stossel 2020 [70] Germany | RCT December 2015 - September 2018 | | | Intervention: N=16, 10.6 (5.19);4.1-17.3 | | M: 10 (62.5%) F: 6 (37.5%) | | NR | | Leukemia/T-cell-lymphoma 8  CNS-tumour 2  Others 6 | | Supervised exercise program | | | mean 7.3 (3.7SD) weeks from diagnosis  6-8 weeks | | | Pre Post (6-8weeks) | | | | Physical function: 6MWT, Knee strength, Arm flexion strength Anthropometry: Weight, BMI, BIA phA (whole body, arm, leg) Fatigue: PedsQL MFS QOL: HRQOL (KINDL) Physical activity: PA level score 1-10 (qualitative interview) | | | | | |  |
|  |  |  |  | Control: n=17, 11.4 (4.25); 5.1-17.7 | | M: 10 (58.8%) F: 7 (41.2%) | | NR | | Leukaemia/T-cell-lymphoma 7 -CNS-tumour 2 Others 8 | | Control: standard care | | | mean 5.9 (3 SD) weeks from diagnosis | | |  | | | |  |  |  |  |  |  |  |
| Taguchi 2018 [137] Japan | Retrospective cohort study January 2016 - December 2016 | | | n=25, 7.15; 8m-14y | | M: 12 (48%) F: 13 (52% | | NR | | CNS tumour 26.9%, RMS 23.1%, Leukaemia 15.4%, Osteosarcoma 7.7%, NBL 7.7%, Lymphoma 7.7%, Renal cell carcinoma 3.8%, nephroblastoma 3.8%, EWS 3.8% | | Multidisciplinary rehabilitation | | | During treatment (mean 54.8 days post diagnosis; 4-228 days) | | | Admit Discharge | | | | Physical function: Functional independence measure score Clinical: Length of stay, type of rehabilitation | | | | | |  |
| Thygeson 2010 [138] USA | Multicentre cohort study NR | | | Children: n=11, 8.55 (1.75)  Adolescents: n=5, 15.4 (1.82) | | Children  M: 7 (64%) F: 4 (36%)  Adolescents  M: 3 (60%)  F: 2 (40%) | | Children  Caucasian 8 (73%) Black 1 (9%) Hispanic Latino 1 (9%) Asian Multiracial 1  Adolescents:  Caucasian 4 (80%) Asian 1 (20%) (9%) | | Children  ALL 2 (18%) Relapsed ALL 2 (18%) Solid tumour 3 (27%) CNS tumour 3 (27%) Sickle cell disease 1 (9%)  Adolescents: ALL 2 (40%) AML 1 (20%) Solid tumour 1 (20%) CNS tumour 1 (20%) | | Yoga | | | During treatment: Time since diagnosis (months) 8.25 ± 7.02 1x 45minute class | | | Pre and post class | | | | Anxiety: STAI (spielberger state trait anxiety inventory) Qualitative | | | | | |  |
| Tsao 2022 [111] | Retrospective cohort study January 1 2017 - October 10 2018 | | | n=18, median 4.8; 1.9-17.8 years | | M: 9 (50%) F: 9 (50%) | | Non-Hispanic 11 (61.1) Hispanic 1 (5.6) Unknown 6 (33.3) | | Leukaemia 7 (38.9) Lymphoma 7 (38.9) CNS Tumour 6 (33.3) Sarcoma 1 (5.6) SCID 2 (11.1) | | Inpatient rehabilitation | | | During treatment 2 weeks | | | Pre and post | | | | None of interest | | | | | |  |
| Winter 2013 [83] Germany | Prospective cohort study  July 2006 - July 2008 | | | Intervention: N=16, 13.5 (2.7) | | M: 7 (43.8%) F: 9 (56.3%) | | NR | | Osteosarcoma 11 EWS 5 | | Inpatient exercise program | | | Post surgery 6 months | | | Post surgery:  6 weeks 3 months 6 month  12 months  18 months | | | | Physical activity : Volume of activity (gait cycles), per day, per hour, intensity per minute. | | | | | |  |
|  | Control: different institution | | | Control: n=15, 14 (3.6) | | M: 9 (60%) F: (40%) | | NR | | Osteosarcoma 10 EWS 5 | |  | | |  | | |  |  |  |  |  | | | | | |  |
| Wurz 2014 [92] Canada | Prospective cohort study | | | n=8, 11.88 (4.26) | | M: 4 (50%) F: 4 (50%) | | NR | | Osteosarcoma 1, CNS 2, ALL 2, CML 1, lymphoma 1, neuroendocrine tumour and Burkitt’s lymphoma 1 | | Yoga classes | | | NR  12 weeks | | | Baseline 12 weeks | | | | QOL: PedsQL 4.0 Physical function: TUG 3m, reach test, ankle DF ROM Physical activity level: GLTEQ | | | | | |  |
| Zardo 2022 [140] Italy | Prospective cohort study  April 2017 - July 2020 | | | n=97, 10.58 (4.5) | | M: 52 (53.6%) F: 45 (46.4%) | | NR | | ALL Lymphoma AML | | Precision-based exercise program | | | >4 weeks from diagnosis  11 weeks | | | T0 T2 | | | | Physical function: TUDS, 6MWT, quads 5RM, yoyo AD | | | | | |  |
| Bogg 2015 [94] Sydney, Australia | | Prospective cohort study January 2012 - October 2012 | n=14, 11 years; 6-17 | | M: 8 (57.1%) F: 6 (42.9%) | | NR | | ALL: Relapse 6, HR 2  AML 2, AA 2, ALCL 1, MPD 1 | | Supervised exercise intervention | | | On admit for HSCT  Until discharge (mean 44 days) | | | | | | Baseline (2 weeks pre admit), 6 weeks post HSCT or on discharge | | | Physical function: 6MWT, balance, leg dynamometer strength, HGS Physical activity: Minutes/week Fatigue: PedsQL MDF  QOL: PedsQL | | | | | |
| Chamorro-vina 2010 [89] Spain | | Prospective cohort study 2007-2008 | Intervention: n=7 8 (4) | | M: 5 (71.4) F: 2 (28.6) | | NR | | B-cell ALL 4 (57%), NBL 1 (14.3%), RMS 1 (14.3%), T cell ALL 1 (14.3%) | | Supervised exercise intervention | | | Admit for HSCT  3 weeks | | | | | | Baseline (preconditioning), post HSCT day +30 | | | Anthropometry: BMI, Weight, Body fat, FFM Physical function: Half squat, bench press, resting Heart rate,  Clinical: Engraftment kinetics, | | | | | |
|  |  |  | Control: n=13 7 (3) | | M: 9 (69.2%) F: 4 (30.8%) | | NR | | B-cell ALL 8 (61.5%), AML 5 (38.5%) | | Historical control | | |  |  |  |  |  |  |  |  |  |  |  |  |  |  |  |
| Diorio 2015 [95]  Canada  NCT02105389 | | Prospective cohort study  January - October 2013 | n=11, median 14; 7.7-16.4 | | M: 6 (54.6%)  F: 5 (46.4%) | | NR | | Leukaemia/ lymphoma 8 (72.7)  Solid tumour 1 (9.1)  Brain tumour 1 (9.1) AA 1 (9.1) | | Yoga | | | During intensive treatment/admit for HSCT  3 Weeks | | | | | | Baseline  day 7  day 14 , day 21 | | | Fatigue:  PedsQL MFS  QOL:  PedsQL 4.0 cancer modules  Qualitative: PA level | | | | | |
| Huang 2023 [71]  China | | RCT  July 2017 to December 2018 | Intervention: n=23, 11.52 (2.48) | | M: 9 (39%) F: 14 (61%) | | NR | | AML: 21 (91%) ALL: 2 (9%) | | Exercise intervention | | | Admission for HSCT,  until discharge | | | | | | Baseline (admission), +14 days from transplant, discharge | | | Fatigue: PedsQL MFS  QOL: PedsQL 3.0 cancer module  Clinical  Engraftment kinetics, supportive care, complications, GvHD, hospital LOS. | | | | | |
|  |  |  | Control: n=21, 12.14 (2.90) | | M: 13 (62%) F: 8 (38%) | | NR | | AML: 9 (43%) ALL: 10 (48%) CML: 2 (9%) | | Usual care | | |  | | | | | |  |  |  |  |  |  |  |  |  |
| Schechter 2023 [72]  Canada | | RCT | Intervention: n=62  8–10y: 24 (38.7) 11–14y: 25 (40.3) 15–18y: 13 (21.0) | | M: 36 (58.1%)  F: 26 (41.9%) | | NR | | Leukaemia 35 (56.5) Lymphoma 10 (16.1) Solid tumour 4 (6.5) Brain tumour 1 (1.6) Not oncology 12 (19.4) | | Yoga intervention | | | -1 to +4 days of HSCT conditioning,  3 weeks | | | | | | Baseline, day 10, day 21 | | | Fatigue: PedsQL MFS | | | | | |
|  | |  | Control n=63,  8–10: 27 (42.9) 11–14: 20 (31.7) 15–18: 16 (25.4) | | M: 39 (61.9%)  F: 24 (38.1%) | | NR | | Leukaemia 36 (57.1) Lymphoma 9 (14.3) Solid tumour 4 (6.3) Brain tumour 1 (1.6) Not oncology 13 (20.6) | | iPad control | | |  | | | | | |  | | |  | | | | | |
| Sen-Malashonak 2019 [73]  Frankfurt, Germany  [134] | | Randomised controlled trial January 2011-December 2014 | Intervention: n=35, median 11.0; 5-17 | | M: 25 (71.4%) F: 10 (28.6%) | | NR | | Leukaemia 22, MDS 3, NBL 2, Haematological disease 5, Soft tissue sarcoma 1, Lymphoma 2 | | Exercise intervention | | | Admit for HSCT  5 sessions weekly during their inpatient period | | | | | | Hospital admission and day of discharge | | | Physical function: 6MWT, VO2 peak, Knee extension dynamometer strength, HGS Other: QOL (KINDL QOL)  Clinical: Complications, GvHD, engraftment kinetics | | | | | |
|  |  |  | Control: n=35, median 12; 6-18 | | M: 23 (65.7%) F: 12 (34.3%) | | NR | | Leukaemia 15, MDS 4, NBL 2, Haematological disease 5,  Soft tissue sarcoma 4, Lymphoma 3,  Nephroblastoma 1, NPC 1 | | Control - mental and relaxation training | | | 5 sessions weekly during their inpatient period | | | | | | Hospital admission and day of discharge | | |  |  |  |  |  |  |
| Morales 2020 [84]  Spain | | Retrospective cohort study June 2013 - June 2018 | Intervention: n=65, 11 (4) ;5- 18 | | M: 63% F: 37% | | NR | | ALL 68%  Other leukaemia’s 32% | | Physical activity | | | Admit for HSCT Until neutrophil engraftment (~3 weeks) | | | | | | 6 years | | | Clinical: Mortality, GvHD, engraftment kinetics, supportive care, toxicities, infections. | | | | | |
|  |  |  | Control: N=53, 10 (4); 4-18 | | M: 64% F: 36% | | NR | | ALL 70% Other leukaemia’s 30% | | Control - self selected | | |  |  |  |  |  |  |  |  |  |  |  |  |  |  |  |
| Jacobs 2021 [74]  Canada  & USA | | Multicentre RCT  June 2016-April 2017 | Intervention: n=3 | | M: 2 (33.3%) F: 4 (66.7%) | | NR | | NR | | Yoga | | | Admit for HSCT 21 days | | | | | | Baseline (day 0), day 10, day 21 | | | Fatigue: PedsQL MFS  QOL: PedsQL 3.0 acute cancer module | | | | | |
|  |  |  | Control: n=3 | |  |  | NR | | NR | | iPad control | | |  | | | | | |  |  |  |  |  |  |  |  |  |
| Rossi 2022  [85]  Italy  NCT03842735 | | Non-RCT pilot May 2016- 26 February 2020 | Intervention: N=36, median 13.75 years | | Check later, not clear based on paper | | NR | | ALL 21, AML 6, Lymphoma 10, solid tumour 2, other haematological disease 8, bone tumours 2. | | Physical activity during HSCT | | | Admit for HSCT  Duration of admission | | | | | | Baseline and discharge | | | Physical function: TUG, gross motor function, flexibility, ankle DF (Passive), knee extension dynamometer strength,  Fatigue: PedsQL MFS | | | | | |
|  |  |  | Control: n=13, median 11.75 years | |  | |  | |  |  | Rehabilitation counselling control | | |  | | | | | |  | | |  |  |  |  |  |  |

ALL: acute lymphoblastic lymphoma; AML: acute myeloid leukaemia; BF%: body fat percentage; BMD: bone mineral density; BMI: body mass index; BOT-2: Bruininks-Oseretsky Test of Motor Proficiency, 2nd Edition; CML: chronic myeloid leukaemia; CNS: Central nervous system; DF: dorsiflexion; Diorio 2015: 54.6% of participant HSCT; EWS: Ewing sarcoma FACIT: Functional Assessment of Chronic Illness Therapy; FFM: fat free mass; FM: fat mass; GLTEQ: Godin leisure time exercise questionnaire; GvHD: Graft versus host disease; HGS: Hand grip strength; HL: Hodgkin’s lymphoma; HRQOL: health related quality of life; HSCT; Hematopoietic stem cell transplant; KINDL: Kinder Lebensqualität fragebogen questionnaire; LM: lean mass; LOS: length of stay; MABC-2: Movement assessment battery for children, version 2; MBL: Medulloblastoma MRD: minimal residual disease; MSAS: memorial symptom assessment scale; MVPA: moderate vigorous physical activity; NBL: Neuroblastoma; NPC: nasopharyngeal carcinoma PA; physical activity; PedsQL MFS: Pediatric quality of life multidimensional fatigue scale; PROM: Patient-Reported Outcomes Measures; QOL; Quality of life; RMS: rhabdomyosarcoma; ROM: range of motion; TUG: Timed up and go; TUDS: time up and down stairs; VSP-A: Vécu et Santé Perçue des Adolescents questionnaire; 6MWT: 6 minute walk test; 9MWR: 9 minute walk run

Supplementary Table S4. Physical activity interventions

| Author (year) | Type | | Diagnoses | | Provider | | Setting (supervision) | | | Frequency, session duration | | Intensity | | Type | | Time | | Follow up | |
| --- | --- | --- | --- | --- | --- | --- | --- | --- | --- | --- | --- | --- | --- | --- | --- | --- | --- | --- | --- |
| Cox 2018 [56] | Physical activity program | | ALL | | APN and PT | | Home-based (parent supervision), OP nurse + PT visits | | | 5×/week, 30 mins  APN: 2x/wk 1-4wks, 1x/week 5-8 weeks, monthly 9-135 weeks.  PT: 1x/wk 1-4wks, 1x/fortnight 5-8 weeks, monthly 9-135weeks | | NR | | Resistance, Aerobic | | 2.5 years | | T0: baseline t1: 6-7wks t2: 8-9wks post induction t3: 135 weeks | |
| Eshbenshade 2014 [102] | Home based exercise | | ALL | | Exercise coach | | Home-based | | | 6x/week, 30-45 minute  1x/week phone call  1x/month clinic review | | NR | | Aerobic, flexibility, resistance | | 6 months | | Baseline and 6 months (end of intervention) | |
| Gohar 2011 [93] | Individualised home exercise program | | ALL | | Physical therapist | | Home based (unsupervised)  Supervised during IP admissions | | | 5x/week, 10-30mins aerobic, Other NR  3x/week when IP | | NR | | Flexibility, resistance, aerobic | | 6-7 months | | End of induction, consolidation, interim maintenance and delayed intensification | |
| Hartman 2009 [57] | Exercise program | | ALL | | Paediatric physiotherapist | | Home-based OP | | | 1-2x/day  Follow up every 6 weeks | | NR | | Flexibility, Resistance, Aerobic | | 2 years | | Diagnosis, 32 weeks, 1 year, 2 years (EOT), 3 years (EOT + 1 year) | |
| Hooke 2016 [105] | Physical activity (Fitbit) | | ALL | | Study nurse, physical therapist Fitbit | | Home-based | | | Daily emails | | NR | | Aerobic | | 2 weeks | | Baseline, -14 days, -7 days, -1 day, +5 days | |
| Kashaul 2013 [58] | Aerobic exercise vs relaxation techniques | | ALL | | Caregiver and professional | | In hospital (supervised) | | | Once daily, 5x/week | | NR | | Aerobic | | 3 weeks | | Pre and post (3 weeks) | |
| Khodashenas 2017 [59] | Aerobic exercise program | | ALL | | NR | | NR | | | 3x per week, 60-minute sessions | | 60-85% max HR | | Aerobic | | 12 weeks | | Baseline and 12 weeks | |
| Marchese 2004 [60] | Physical therapy | | ALL | | Physical therapist | | Outpatient Home based | | | 1:1 session at baseline, week 2, 4, 8 and 12. Home plan for 3-5 days a week. | | NR | | Aerobic, resistance, flexibility | | 4 months | | Pre and post (4 months) | |
| Masoud 2023 [61] | Exergaming | | ALL | | Researcher | | In hospital (supervised) | | | 2x/week (60-minute sessions) | | Moderate intensity (50-70% increase in pred HR max | | Aerobic exergaming | | 3 weeks | | T1: baseline  T2: Post 1 week T3: Post 3-week intervention | |
| Moyer-Mileu 2009 [51] | Home-based exercise and nutrition program | | ALL | | Registered dietitian | | Home-based (unsupervised) | | | Minimum 3x/week, 15-20mins  Monthly review of activity and nutrition | | Moderate to vigorous | | Aerobic, muscular activity, flexibility, sports | | 12 months | | Baseline, month 3, month 6, month 9, month 12 | |
| Orgel 2021 [49] | Nutrition counselling and exercise program | | HR ALL | | Physiotherapist | | Home-based | | | Daily, 15-30 mins  Weekly adjustment of individual goals | | Moderate | | Aerobic, resistance | | 4 weeks | | Baseline, 4 weeks | |
| San Juan 2007 [91] | Exercise program | | ALL | | Exercise physiologists and qualified fitness instructors | | Outpatient | | | 3x/week, 90-120 minutes | | 50% HRmax 10 minutes, to ≥70% HRmax 30 minutes | | Aerobic, Resistance, Flexibility | | 8 weeks | | Baseline Post training (8 weeks) | |
| San Juan 2007 [97] | As above | | As above | | As above | | As above | | | As above | | As above | | As above | | 16 weeks | | Baseline Post training (16 weeks) Detraining | |
| Tanner 2017 [100] | Physical therapy | | ALL | | Physical therapist | | Outpatient | | | Individualised | | Individualised | | Flexibility, resistance, balance, gait | | N/A | | Baseline, end of IM, end of DI, maintenance cycle 1, 3 and 6. | |
| Waked 2018 [62] | Exercise program | | ALL | | Researcher | | Outpatient | | | 2x/week for 0-6m and 1 session/week for 6-12m | | 3-6 RPE (out of 10) | | Aerobic, Resistance, Flexibility | | 12 months | | Baseline, 6 months, 12 months | |
| Wright 2003 [86] | Ankle ROM physical therapy | | ALL | | Physical therapist | | Outpatient | | | 1x/week | | NR | | Aerobic, resistance, flexibility | | 2 years | | Not specified | |
| Yeh 2011 [75] | Aerobic Program | | ALL | | Videos | | Home-based (unsupervised) | | | 3x/week, 30 minutes | | 40-60% HRR | | Aerobic | | 6 weeks | | Baseline, weekly for 1-6 weeks. 1 month post intervention. | |
| Zhang 2019 [50] | Healthy eating and active living | | ALL | | Lifestyle coach and Self-guided | | Home-based (unsupervised) | | | Daily, >60 minutes  >3x/week “Bone strengthening activities”  Weekly phone calls | | NR | | NR | | 12 weeks | | Baseline, 6 months | |
| Atkinson 2023 [101] | Robotic rehabilitation | | Mixed | | Exercise physiologist | | Outpatient (supervised) | | | 2x/week, 20-40 mins | | NR | | Advanced robotic gait training device | | 6 weeks | | 0 and 6 weeks | |
| Caru 2020 [106] | Home based physical activity program | | Mixed | | Exercise physiologist | | Home-based telehealth (supervised) | | | 2x/week, 45 minutes | | Moderate intensity | | Aerobic + flexibility + resistance + balance | | 6 weeks | | 0 and 6 weeks | |
| Hooke 2019 [88] | Physical activity | | Mixed | | Nurse practitioner | | Home-based (unsupervised)  Outpatient | | | Individualised  Monthly OP visit | | Personalised | | NR | | 4 months | | 2 months, 4 months and 6 months | |
| Grimshaw 2024 [98] | Behaviour change physical activity program | | Mixed | | Study physical therapists | | In hospital/OP/ videoconference | | | Daily step goal 1x/week, 1 hour session | | NR | | NR | | 10 weeks | | Baseline, post intervention | |
| Bluth 2016 [135] | Martial arts | | Mixed | | Martial arts instructor | | NR | | | NR 1-hour sessions | | NR | | Martial arts | | 12-month program | | Pre and post session | |
| Fukuhara 2020 [139] | Yoga session | | Mixed | | Yoga teacher/ researcher | | In hospital | | | 1 session (1:1), 20-60 minutes | | Individualised | | Yoga | | 1 session | | Pre and post session | |
| Tsao 2022 [111] | Intensive rehabilitation | | Mixed | | MDT | | In hospital | | | 5x/week, 2-3h of PT, OT and/or SLT | | NR | | NR | | 2 weeks | | Pre and post | |
| Corr 2017 [87] | Physical activity (Prehabilitation) | | Sarcoma | | Physical therapist In person, supervised | | Inpatient then outpatient | | | 3 times a week (30-60 mins each | | 50-70% HR max | | Aerobic Resistance Flexibility | | 10-12 weeks | | Baseline, pre surgery (10-12wks), post-surgery (20-22wks) | |
| Dalla Santa 2023 [103] | Individualised physiotherapy program | | Mixed | | Physiotherapist In person supervised and independent | | Outpatient, home based | | | 1-3x/week supervised, 45-60 mins, 3x/week independent or parent supervision (15-30 mins) | | 5-6 RPE (Moderate) | | Aerobic, strengthening, stretching, balance | | 8 weeks | | Pre and post | |
| Gaser 2022 [64] | RCT  September 2017 - June 2020 | | Haematological | | Exercise physiologist' | | In hospital and over zoom (during pandemic restrictions) | | | 2-3 supervised sessions/week, 30 minutes | | Not predefined | | Aerobic, resistance, flexibility, relaxation | | Mean 7.1 months (intensive treatment) | | V0: baseline  (V1) end of induction  (V2) end of consolidation  (V2) end of intensive treatment | |
| Gotte 2018 [77] | Home-based exercise program | | Mixed | | Study coordinator provided plan - not supervised | | Home-based | | | Individual plans | | individual goals | | Aerobic Resistance Co-ordination | | 6-8 weeks | | T1: baseline T2: week 6-8 | |
| Govardhan 2019 [107] | Yoga | | Brain tumours | | Yoga instructor | | In hospital | | | 3x/week (offered 5x), 1 hour | | Low intensity | | Yoga | | 4 weeks | | Baseline 4 weeks | |
| Hamari 2019 [65] | Active video game | | Mixed (non-CNS) | | Physical therapist (not supervised) | | Hospital/home based | | | Daily (30mins) | | Light to moderate | | Aerobic | | 8 weeks | | Baseline, 2 months, 6 months, 12 months, 30 months | |
| Hayati 2019 [78] | Aerobic exercise | | Mixed | | Study personnel (non-supervised) | | In hospital | | | Daily, 15 minutes | | NR | | Aerobic relaxation | | 5 days | | Day 1, day 5 | |
| Hinds 2007 [66] | Enhanced physical activity | | Mixed | | Study team member (supervised) | | In hospital | | | 2x/day, 30mins | | NR | | Aerobic | | 2-4 days | | Day 0, day 1, day 2, day 3 | |
| Fuiza-luces 2017 [63]  Fuiza luces 2017 [132] | Exercise intervention | | Mixed | | Fitness instructor | | Hospital gym | | | 3x/week, 60-70mins | | 60-70% HR max | | Aerobic, resistance | | mean 19 weeks | | Treatment initiation, end of treatment (approx. 20 weeks), 2 months (8 weeks) post end of treatment. | |
| Morales 2020 [99] | Exercise intervention | | Mixed | | Exercise physiologist' | | Hospital gym | | | 2-3x/week, 60-70 mins | | 65-85% HR reserve | | Aerobic, resistance | | Duration of neoadjuvant/ intensive treatment, median 22 weeks | | Baseline (dx), end of neoadjuvant treatment (solids) or intensive treatment (leukaemia), 1,2,3,4,5 years post diagnosis | |
| Muller 2014 [79] | In hospital exercise intervention | | Bone tumours | | Sports scientist | | In hospital | | | Every second day (offered daily Monday-Friday), 15-45mins | | 13-16 Borgs rating | | Balance flexibility resistance | | Duration of treatment | | Baseline, post local control, treatment completion (6m later), 6m post treatment completion | |
| Fridh 2023 [80, 96, 104] | Exercise and social support | | Mixed | | Classmate ambassadors and | | In hospital | | | During inpatient admission: 3x/week (5-30 mins) 2x/week (30-120 mins) group sessions | | mean HRmax 69.3% (68.1-70.4) | | Aerobic, resistance, balance, | |  | | Baseline (diagnosis)  3 months  6 months  1 year post treatment | |
| Orsey 2017 [109] | Yoga | | Mixed | | Yoga instructor (1:1, or group) | | Inpatient/ outpatient | | | Minimum 4x/8weeks, 60 minutes | | NR | | Yoga | | 8 weeks | | Baseline 8 weeks | |
| Oschwald 2023 [136] | Whole-body vibration training and exercise program | | Mixed | | Exercise physiologist/ physiotherapist | | In hospital (during admissions) | | | 3x/week | | NR | | Aerobic Resistance Flexibility Vibration | | Variable, 7-52 weeks | | N/a | |
| Ouyang 2019 [81] | Group physical activity | | Mixed | | Researcher/ researcher assistant | | In hospital | | | ~1x/week, 30-40mins sessions  (12 sessions total) | | light intensity | | Flexibility resistance | | 3 months (12 weeks) | | Baseline, week 3, week 6, week 9, week 12 | |
| Platschek 2017 [90] | Active video game | | mixed | | sports scientist (supervised) | | Inpatient and outpatient during neoadjuvant chemo | | | 6x/week, 45 mins | | individually set | | Aerobic resistance | | 12 weeks | | Baseline, 4 weeks, 8 weeks 12 weeks | |
| Pouplier 2024 [142] | Structured active play | | NR | | Exercise professionals (supervised) Parent led at home | | In hospital Home-based | | | 3x/week group, 45mins Daily home sessions | | NR | | Gross motor | | 6 months | | NR | |
| Saultier 2021 [69] | Physical activity program | | Mixed | | Adapted physical activity instructors | | In hospital, additional locations | | | 30 sessions, 30 - 90mins. 25 multi-activity sessions 90 -240 mins | | 60-70% HRmax | | Resistance aerobic balance flexibility | | 6 months | | T0: baseline 6 months 12 months | |
| Sonoda 2023 [82] | Physical therapy | | Haematological | | Physical therapist 1:1 | | In hospital | | | 5x/week, 30-60mins | | 11-13 borgs scale | | Aerobic, resistance, flexibility, balance | | 40 weeks | | At admission (baseline): week 0. After initial treatment: week 8. At discharge: week 40. | |
| Stein 2019 [108] | Yoga | | Mixed | | Yoga instructor | | Via skype In hospital/ outpatient | | | 1x/week, 45mins | | Low-moderate intensity postures | | Yoga | | 10 weeks | | Baseline week 5 week 10 | |
| Stossel 2020 [70] | Exercise program | | Mixed | | Exercise scientist (supervised) | | Inpatient, outpatient, at home | | | 3x/week, 45-60mins | | Moderate intensity: 60-75% HRmax, 12-13 Borg scale | | Aerobic Resistance | | 6-8 weeks | | Pre Post (6-8weeks) | |
| Taguchi 2018 [137] | Multidisciplinary rehabilitation | | Mixed | | PT, OT, SLT | | Inpatient | | | 5x/week (weekdays) | | NR | | Individualised | | LOS mean 71.8 days (range 13-236) | | Admit Discharge | |
| Thygeson 2010 [138] | Inpatient yoga class | | Mixed | | Yoga teacher (researcher) | | In hospital | | | Once | | NR | | Yoga | | Single yoga class | | Pre and post class | |
| Winter 2013 [83] | Adjuvant exercise intervention | | Bone tumours | | Sports scientist (supervised) | | In hospital | | | Daily (while inpatient) 30-60 minutes | | Individualised | | Aerobic Resistance Flexibility | | 6 months | | Post surgery: 6 weeks, 3 months, 6 months, 12 months, 18 months | |
| Wurz 2014 [92] | Yoga classes | | mixed | | Yoga instructor | | Outpatient | | | 2x/week group session, 60 minutes | | NR | | Yoga | | 12 weeks | | Baseline 12 weeks | |
| Zardo 2022 [140] | Precision based exercise program | | Haematological | | Supervised (not specified) | | No specified | | | 3x/week (duration dependant on ITR) | | Variable based on intensity of treatment | | Aerobic Resistance Flexibility Neuromuscular | | 11 weeks | | T0: Pre T1: Post | |
| Kowaluk 2022 [67] | Interactive video games physical activity | | Leukaemia | | Researcher (1:1) | | In hospital | | | 3x/week, 20 minutes | | Moderate intensity Session 1-4: 70% HRpeak Session 5-8: 75% HRpeak Session 9-12: 80%Hrpeak | | Aerobic video game | | 4 weeks | | Baseline 4 weeks  14 months | |
| Lam 2018 [68] | Home-based training program | | Mixed | | Nursing student (1:1) | | Home-based | | | 2x/week for 4wks then 1x/week for 20 weeks, 1 hour | | Low to moderate | | Aerobic Resistance Flexibility Relaxation | | 6 months | | Baseline 6 months 9 months | |
| Bogg 2015 [94] | Supervised exercise intervention | Mixed | | In person, individually supervised | | Accredited exercise physiologist | | In hospital | 5 days/week (offered twice daily), (10-60 min sessions) | | Low to moderate Mean RPE 3.7/10 | | Aerobic Resistance Flexibility | | Until discharge | | Baseline (2 weeks pre admit) 6 weeks post HSCT or on discharge | |  |
| Chamorro-vina [89] | Supervised exercise intervention | Mixed | | In person, individually supervised | | Fitness instructor | | In hospital | 5 days/week, ~50 minutes each | | Low to moderate 50-70% HRmax | | Aerobic | | Start of HSCT conditioning to neutrophil engraftment (~3 weeks) | | Baseline (preconditioning) Post HSCT day +30 | |  |
| Diorio 2015 [95] | Yoga | Mixed | | In person individually supervised | | Yoga teacher | | In hospital | 3x/week (offered 4-5), NR | | NR | | Yoga | | 3 weeks | | Baseline, day 7, day 14, day 21 | |  |
| Huang 2023 [71] | Exercise intervention | Mixed | | In person, individually supervised | | Exercise physiologist | | In hospital | 3 days/week, 30-40 minutes each | | Moderate 12-14 RPE (Borgs scale) | | Aerobic Resistance Flexibility | | Admit to discharge | | Baseline (admission), +14 days from transplant, discharge | |  |
| Jacobs 2021 [74] | Yoga intervention | Leukaemia & lymphoma | | In person, individually supervised | | Yoga instructor | | In hospital | 5 day/week, 15-45 minutes each | | NR | | Yoga | | 3 weeks | | Baseline, day 10, and day 21. | |  |
| Morales 2020 [84] | Exercise intervention | Leukaemia | | In person, individually supervised | | Fitness specialist | | In hospital, patient room | 5 day/week, ~60mins each | | Moderate, 65-85% HR max | | Aerobic Resistance | | Until neutrophil engraftment (~3 weeks) | | 6-year retrospective review | |  |
| Schecter 2023 [72] | Individualised yoga program | Mixed | | In person, supervised | | Yoga instructor | | In hospital | 5x/week, 15-45mins | | Low, moderate and high | | Strength, flexibility balance | | 3 weeks | | Baseline: day 0 day 10  day 21 | |  |
| Sen-Malashonak 2019 [73, 134] | Exercise intervention | Mixed | | In person, individually supervised | | NR | | In hospital | 5 day/week, 30- 60 mins each | | Moderate to hard, RPE 12-14 (Borgs scale) | | Aerobic Resistance Flexibility | | Duration of admission | | T1: Admission T2: Discharge | |  |
| Rossi 2022 [85] | Exercise intervention | Mixed | | In person, individually supervised | | DNPT 3/5 sessions Parents 2/5 sessions | | In hospital, patient room | 5 day/week, 30 mins each session | | Light to moderate 2-3/10 (Borg scale) | | Aerobic Resistance Flexibility | | Duration of admission | | Baseline and discharge | |  |

ALL: Acute lymphoblastic leukaemia; DI: delayed intensification; EOT: end of treatment; HR: heart rate; HRR: heart rate reserve; IM: Interim maintenance; ITR: Intensity treatment rating; MDT: multi-disciplinary team; NR; not reported; OT: occupational therapist; PT: physical therapist; RPE; rate of perceived exertion; SLT: speech language therapist.

Supplementary Table S5. Dietary results

|  | **Negative** | **No effect** | **Positive** |
| --- | --- | --- | --- |
| **Calorie intake** | | | |
| Cohort | 0-1m +47.4% p=0.0002 [46]  0-12m +21.7%, p=0.033 [48] | 0-3m -12%, p=0.105 [45]  0-6m -8.7%, p=0.17 [50]  0-6m +24.1% p=0.25 [46] |  |
| RCT |  | 0-12m IG -25.9% p=0.0522,  CG -5.2% p=0.625 [47] |  |
|  | Mean of multiple time points only [51] | | |
| **Diet quality** | | | |
| Cohort |  | HDI 0-12m p=0.065 [48]  HEI 0-6m +3.14, p=0.29 [50]  GL 0-6m: -2g/1000kcal, p=0.052 [46] | DQI 0-12m +5.22, p=0.003 [48]  GL 0-1m: -4g/1000kcal p=0.012 [46] |
| **Fruit & vegetables** | | | |
| Cohort |  | Fruit 0-6m p=0.68; vegetables 0-6m p=0.58 [50]  Fruit & vegetables 0-6m p=0.22 [46] | ↑Fruit^a^ 0-3m p=0.005; ↑vegetables 0-3m ^a^ p=0.045 [45]  ↑Fruit & vegetables 0-1m p=0.005. Vegetables 0-1m p=0.0003 , 0-6m p=0.033 [46] |
| **Macronutrients** | | | |
| Cohort |  | %kcal CHO 0-6m -4.52, p=0.05 [50]  %kcal fat 0-6m +1.53, p=0.39 [50]  Protein (g) 0-3m -7.5%, p=0.271 [45]  CHO (g) -2.2%, p=0.602  Protein p=0.97; CHO p=0.63, Fat p=0.68 [48]  %kcal CHO 0-6m -1%, p=0.064 [46] | %kcal Protein 0-6m ↑2.54%, 0.04 [50]  CHO (g) 0-3m ↓20%, p=0.049 [45]  %kcal Protein 0-1m +2% p=0.042; 0-6m +1%, p=0.027 [46]  %Kcal CHO 0-1m -5%, p=0.002 [46] |
| ^a^: Food frequency questionnaire; HDI: healthy diet index; HEI: healthy eating index; DQI: diet quality index; GL: glycaemic load g/1000kcal (median); m: months; CHO: carbohydrates; %kcal: percent of calorie intake | | | |

Supplementary Table S6. Anthropometry and body composition results

| **Nutrition interventions** | | | | | | | |
| --- | --- | --- | --- | --- | --- | --- | --- |
| **Body composition** | | Negative | | | No change | | Positive |
| Cohort | |  | | | zTSFT +0.75 p=0.1 [48]  zMUAC 0.23 p=0.51 [48] | |  |
| Non-RCT | |  | | | Median BF% 0-1m: IG +5.1%, CG +10.7%, p=0.13 [49] | | Median BF% 0-1m OW/OB IG +1.5%, CG +9.7%, P=0.02 [49] |
| **BMI** | | | | | | | |
| Cohort | | zBMI: 0-12m +0.5 (0.88) p=0.002 [48] | | | zBMI: 0-6m +0.02 p=0.93 [50]  zBMI: 0-1m values NR p=0.419 [46] | |  |
| Non-RCT | |  | | | zBMI: 0-12m IG +0.58, CG +0.85: p=0.08 [44] | |  |
| RCT | |  | | | BMI% IG +4.81, CG +0.16, p=1 [47] | |  |
|  |  | IG +0.18 (-0.82, 0.47), CG +0.29 (-0.89, 0.31) [51] | | | | | |
| OW/OB: overweight/obese; +: increase; -: decrease; IG: intervention group: CG: control group; BMI%: body mass index percentile: zBMI: body mass index z-score; | | | | | | | |
| **Physical activity interventions** | | | | | | | |
| **BMI** | | Negative | No effect | | | | Positive |
| Cohort | |  |  | | | |  |
|  |  | %BMI -4.2 p=NR [102] | | | | | |
| Non-RCT | |  |  | | | | BMI: IG +1.6, CG -0.6; p=0.001 [89] |
| RCT | |  | %BMI IG +16, CG +13: p=0.75 [56]  BMI IG +0.5, CG +0.5: p=0.853 [63]  BMI: IG +0.4, CG +0.4: P=1 [69]  BMI: IG +0.39, CG +0.01: P=0.416 [70]  zBMI IG +1.53, CG +1.38: P=0.69 0-2y [57] | | | |  |
| **Body composition** | | | | | | | |
| Non-RCT | |  |  | | | | BF^c^ (kg) IG +1.2kg, CG -0.3kg: p=0.006 [89]  FFM^c^ (kg) IG +1.4kg, CG -0.7kg: p=0.021 [89] |
| RCT | |  | zBF% 0-2y IG +1.04, CG +1.56: P=0.25; 2-3y IG -1.08, CG -0.49: P=0.013 [57]  zLBM 0-2y IG -0.61, CG -0.12: P=0.16; 2-3y IG +0.29, CG +0.29: P=0.66 [57]  FM: IG +0.5kg, CG +0.6kg: p=0.7  LM: IG +1.1kg, CG +0.5kg: p=0.1 [69] | | | | Median Bf% 0-6m IG -0.4, CG +1.2: p=0.001^a^; 0-12m IG -1.9, CG +1.7: P=0.001^B^  LBM (kg) 0-6m IG +0.723 p<0.05, CG 0.096: p=0.012^a^; 0-12m IG +2.38 P<0.05, CG +0.099: P=0.001^B^ [62] |
|  |  | Phase angle reported only [70] | | | | | |
| **Bone density** | | | | | | | |
| Non-RCT |  | | | BMC LS: IG -4.5, CG -5.7: P=0.81 [79] | | zLS: IG -0.4, CG -0.8: p=0.04 [79] | |
| RCT |  | | | zLS: IG -0.34, CG -0.16: p=0.61 [56]  TB- IG -0.75 P=0.003, CG -0.96 P=0.0003: P=0.65 0-2y [57]  zLS: IG -0.15, CG -0.04, P=NS 0-2y [57] | | zLS: 0-6m IG +0.051, CG +0.004: p=0.001^a^; 0-12m IG +0.081, CG +0.012: P=0.001^B^ [62]  zTB: 0-6m IG +0.031, CG -0.009: P=0.027^A^; 0-12m IG +0.058, CG -0.017: P=0.001^B^ [62] | |
| ^a^: between groups at 6 months; ^B^: between groups at 12 months: ^c^ estimated; CG: control group; IG: intervention group; BF%:body fat percentage; LBM: lean body mass; FFM fat free mass; FM: fat mass; LM: lean mass; BMC: bone mineral content; LS: lumbar spine; TB: total body; z: z-score; BMI: body mass index; TSFT: tricept skinfold thickness; MUAC: mid-upper arm circumference; %BMI: Body mass index percentile; OW/OB: overweight/obese; y: years; m:months | | | | | | | |

Supplementary Table S7. Physical function results

|  | **Negative** | **No change** | **Positive** |
| --- | --- | --- | --- |
| **6MWT** | | | |
| Cohort |  | ↓ p=0.14 [94] | ↑17.1% p=0.002 [101]  ↑47.9% p=0.015 [103] |
|  | ↑16% p=NR [102]  ↑33.8% p=NR [98] | | |
| RCT |  | ↑19% IG, ↑39.8% CG; p=0.06 [56] | ↑IG +9.9%, ↓CG -0.2%: P=0.001 [61]  ↑IG +15.4%, ↑CG +6.1%: 0-6m p<0.001 [69]  ↓IG -3.8%, ↓CG -19.8%; p=0.002 [73]  ↑IG +9.5%, ↓CG -2.7%: p=0.049 [70] |
| Non-RCT |  | ↔IG p=0.623, ↓CG p=0.006; p=0.06 [82] |  |
| **9MWR** | | | |
| Non-RCT | ↑IG, ↓CG: p=NR [87] | | |
| RCT |  | ↑11.6% IG, ↑0.6% CG; p=0.25 [60] |  |
| **Exercise capacity** | | | |
| Cohort |  | VO2max (ml/kg/min) [99]  VO2peak (ml/min), VO2max (ml/min/kg): leukaemia, lymphoma, solids, CNS [96] [3months] | ↑VO2max (ml/kg/min) p=0.018 [101] |
| Non -RCT |  | Watt max [104] | ↑ VO2max(ml/kg/min) p=0.034; [104]  ↑VO2max (ml/kg/min) p=0.0062; ↑Maxx watt p=0.0038 [80] |
| RCT |  | VO2peak (ml/min) [63] p=0.435^c^  VT (ml/min) p=0.040^c^ [63]  ↓IG, ↓CG: VO2max (ml/kg/min);  ↔VT [73]  VO2 (ml/min) [67] ^d^ | ↑IG p=0.02, ↑CG p=0.34: VO2max (ml/kg/min) [67]^d^ |
| Non-RCT and RCT p-value for group x time unless otherwise stated. Cohort is change over time; VO2p: vo2 peak (ml/minute); VO2max: ml/kg/minute: VT: ventilatory threshold (ml/minute): Watts: max matt (W); ^b^: IG and CG comparison at t2 only; ^c^: significance set at 0.005; ^d^: within group x time analysis (student t test) | | | |
| **TUG** | | | |
| Cohort study | ↓31.6% p<0.05 Leukaemia [96] | ↔ 0% Lymphoma p=ns, ↑14.3 CNS p=ns, ↑11.4% solids p=ns [96] | ↑9.5% P=0.05 [91, 97]  ↑16.8%, p=0.01 [3m] [92] |
|  | ↑ [98] p=NR | | |
| Non-RCT |  | ↓IG 2.5%, ↓CG 16.4% [80] (6months) p=0.4129  Post^a^: p=0.093 [85] |  |
|  | [87] p=NR | | |
| RCT |  | ↑IG 9.5%, ↓CG 2.6%: p=0.104 [3m] [63, 99] |  |
| **TUDS** | | | |
| Cohort study |  | ↑4.8% (8weeks) [91]  ↑7.5% [99] | ↑8.4% p<0.001 (16weeks) [97] |
|  | [98] p=NR | | |
| RCT |  | ↑IG 17.2%, ↑CG 1.3%: p=0.376 [63]  ↑IG 12.7%, ↑CG 5.5% p=0.17 [60] |  |
| **Rise From floor** | | | |
| Cohort |  |  | ↑Floor rise (/4) p=0.001 [103] |
|  | ↑Timed rise from floor [98]; ↑ [100] | | |
| ↑: positive change, a decrease in time; ↓: negative change, an increase in time; ↔ No significant change; [3m]: 3 meter distance version of test; ^a^: analysis post intervention between groups | | | |
| **Sit-to-stand (reps)** | | | |
| Cohort | ↓Leukaemia [96] 0-3m p<0.05 | ↔Lymphoma, CNS, solids [96] 0-3m |  |
|  | ↑ [98] | | |
| Non-RCT |  | 0-6m p=0.5057 [80] |  |
| **Balance** | | | |
| Cohort | ↓FT Leukaemia [96]  ↓SLB seconds p=0.01 [94] | Lymphoma, solids [96]  SLB P=0.839 [103] | ↑FT CNS [96] |
|  | ↑SLB [100] | | |
| Non-RCT |  | ↔ Nielsen [80] |  |
| RCT |  |  | ↑IG, ↑CG: FT p=0.048 [69] |
| **Flexibility: Sit and reach** | | | |
| Cohort |  | ↑ [101] | ↑cm [92] |
|  | ↑42.2% cm [102] | | |
| Non-RCT |  | Post: p=0.490 [85] |  |
| RCT |  |  | ↑cm [69] |
|  | [51] p=NR | | |
| **Ankle DF ROM** | | | |
| Cohort |  | ↑81.4% Passive [102]  ↑ A/P NR [100]  Active (L,R); Passive (L, R) [92] |  |
| Non-RCT |  | Post: Passive [85] p=0.916 |  |
|  | [86] Active and passive | | |
| RCT | IG ↓ 35.9%, CG↓7.53%: Active (L) p=0.03 [110] | Active (R): Passive (L)(R) [56] | ↑47% IG, ↓8.4% CG; Active p<0.01 [60] |
| **Motor performance tests** | | | |
| Cohort | ↑12% Bot-2 [102] p=NR  ↑MABC-2 [98] p= NR  PDMSII or BOT-2 [100] p=NR | | |
| Non-RCT |  | GMFM-ALL [85] |  |
| RCT |  | Bot-2 [56]  MABC-2 [65]  BSID-II/MABC-2 [57] |  |
| **Strength** | | | |
| Cohort |  | ↔ HGS [96]  HGS, KE [94]  KE [85] | ↑6RM: bench press, Lateral row, leg press [91]  ↑5RM bench press, lateral row, leg press [97]  ↑5RM bench press, lateral row, leg press [99]  ↑Back & leg dynamometer [101] |
|  | ↑16.9% HGS; ↑11.1% KE [102] | | |
| Non-RCT |  | ↔ HGS [80] |  |
| RCT |  | HGS, KE [56]  HGS D, KE [73]  AF [70] | ↑5RM Bench press, lateral row, leg press [63]  ↑Medicine ball throw, Myotest [69]  ↑HGS [68]  ↑HGS ND [73]  ↑KE [60]  KF [70] |
| KF: Knee flexion; KE: Knee extension ; HGS: Hand grip strength; D: dominant; ND: non-dominant; RM: Repetition maximum; AF: | | | |

Supplementary Table S8. Physical activity results

|  | Negative | No effect | Positive |
| --- | --- | --- | --- |
| **Physical activity** | | | |
| Cohort |  | ↔ Steps/day ^F^ [105]  ↓Mins/day ^a^ [50]  ↔GLTEQ; ↔Steps/day ^a^ [88]  ↑GLTEQ p=0.249 [101] | ↑MVPAL (MLTPAQ)^C^ [106]p<0.0001  ↑GLTEQ (METs; mins) [92]  ↑AQ [107] p=0.0018 |
|  | ↑Steps/day ^F^ [98] p=NR | | |
| Non-RCT |  | ↑IG p=0.02: Steps/day ^F^ p=0.49 ^b^; ↑IG p=0.04: Active minutes ^F^ p=0.26 ^b^ [77] | ↑MVPA ^C^ (CLASS-C) [81] p=0.001 |
|  | ↑IG, ↑IG: MPA^A^ mins/day; gait cycles ^A^ p=NR [79]  ↑IG, ↑CG: Gait cycles/day ^A^ p=NR [83] | | |
| RCT |  | ↑IG, ↑CG; hours/week ^a^ p=0.73 [56]  ↓IG, ↓CG: MET hr/week ^C^ p=0.38 [65]  ↑IG, ↑CG; MVPA^A^ min/day p=0.6 [63]  ↔GLTEQ [61] | ↑MoMo ^C^ p=0.014 [70]  ↑CUHK-PARCY p=0.001 [68] |
|  | ↑IG, ↑CG: MVPA^A^ p=NR; ↑IG 44%, ↑CG 37%: steps/day ^A^ p=NR [64]  Missing data for time points and significance not reported: Steps/day ^D^; mins/day (AG) [51] | | |
| ^a^ : Accelerometery; ^b^: statical analysis between groups post intervention; ^c^: Questionnaire; ^D^: pedometer; ^F^: FIT BIT; AG: activity gram questionnaire; AQ: activity questionnaire; MET: metabolic equivalent time; GLTEQ: Godin-Leisure-Time Exercise Questionnaire ; CUHK-PARCY: Chinese University of Hong Kong Physical Activity Rating for Children and Youth (Scored 0-10); QR: Qualitative reported; MoMo: German MoMo activity questionnaire; MLTPAQ: Minesota leisure time physical activity questionnaire; CLASS-C: Children’s Leisure Time Activities Study Survey-Chinese | | | |

Supplementary Table S9. Fatigue and quality of life results

|  | Negative | No effect | | Positive |
| --- | --- | --- | --- | --- |
| **Fatigue** | | | | |
| Cohort | ↓FS [88] Solids  ↓FS [105] | PedsQL MFS [94]  FS ALL + lymphoma [88]  PedsQL MFS [85] | | ↑FQ [107]  ↑FACIT [101]  ↑PedsQL MFS p=0.024 [90] |
|  | PedsQL MFS [108] (NR)  PedsQL MFS [95] (NR) | | | |
| Non-RCT |  | AFIC [78]  PedsQL MFS [75] | |  |
| RCT |  | FS [66]  PedsQL MFS/FS-p [72]  ↑IG, ↔CG: PedsQL MFS ^b^ [70]  PedsQL MFS [65]^a^ | | ↑IG, ↑CG; PFS p<0.001 [58]  ↑PedsQL MFS [61]  ↔IG, ↓cg; PedsQL MFS p=0.049 [71]  ↑IG, ↓CG; C-FS p<0.001 [68]  ↔IG, ↓CG: PedsQL MFS ^c^ p=0.013 PR [70] |
| FQ: fatigue questionnaire; AFIC: Allens fatigue in childhood; FS: Fatigue scale; PedsQL MFS: Paediatric quality of life multidimensional fatigue scale; PFS: Piper fatigue scale; C-FS: Chinese version of the fatigue scale; MSAS: Memorial symptom assessment questionnaire, “a lack of energy;” FACIT: The Functional Assessment of Chronic Illness Therapy  Fs-p: Fatigue scale parent; Pr: parent reported; ^a^: parent proxy report for children <10 years; ^B:^ Patient reported; ^c^: parent/proxy reported | | | | |
| **Quality of life** | | | | |
| Cohort |  | | PedsQL [94]  Peds QL 4.0 Pr p=ns [109] | ↑PedsQL 4.0 Pr p=0.03 [103]  ↑EORTC QLQ C30 P=0.018 [101]  ↑Peds QL 4.0 Pt p=0.006 [109]  ↑PedsQL 4.0 Pt + Pr (p=0.02; P=0.03) [92] |
|  | PedsQL Pt + P; PedsQL-CM Pt + Pr; [98] | | | |
| Non-RCT |  | |  | ↑KINDL PT ^B^ [77] |
| RCT |  | | PedsQL-CM 3.0 Pt + Pr [63]  KINDL Pt p=0.504 [70]  IG ↔, ↓CG; P=0.953 PedsQL 3.0 [71]  PedsQL 3.0 Cancer & general: Pt + Pr [60]  ↓IG, ↓CG: KINDL Pt + Pr [73]  +4.9% IG p=0.623, -7.3% CG p=0.018 PedsQL [82] | ↑KINDL-Pr p=0.046 [70]  ↑IG, ↓CG; PedsQL-CM P=0.045 [68]  ↑ VSP-A Pr p=0.04 [69] |
|  | Overall mean for IG+CG combined reported only [74] | | | |
| PedsQL-CM 3.0: Paediatric quality of life cancer module version 3. KINDL: ;Pt: patient reported; Pr: parent reported; ^B^: analysis at t2 between groups; ^c^: group analysis over time | | | | |

Supplementary Table S10. Feasibility of nutrition interventions

| Author (year) | Recruitment | Retention | Participation | Adherence | Adverse events |
| --- | --- | --- | --- | --- | --- |
| Hill 2018 [44] | N/A | N/A | N/A | N/A | NR |
| Li 2016 [47] | NR | NR | NR | NR | NR |
| Moyer-Mileur 2009 [51] | NR | 13/14, (92.9%) | NR | NR | NR |
| Napartuk 2023 [48] | 67.4%, (62/92) | 75.8%, (6.83) | 78.7%, Mean visits 4.72 (1.06 SD) out of 6 | NR |  |
| Orgel 2021 [49] | 90.9%, (40/44) | 97.5%, (39/40) | 86.5% (339 of 392 visits) *(≥80%)* | Diet: 82.1% *(≥75%)*  Exercise: 31.2% *(≥75%)*  *Fitbit flex 2: 59% used the devise (defined as any tracked activity) >50% of days.* | NR |
| Viscardi 2021 [45] | NR | NR | 75.8% | NR | NR |
| Walters 2021 [46] | ~80%, (28/34) | 82.1%, (>70%) | NR | Diet: 60% (SR) | NR |
| Zhang 2019 [50] | 50%, (15/30) | NR | 86.7%, (13/15) (>80%) | NR | NR |

~: estimated value reported by study; NA: not applicable to study design; NR: not reported; *( ):* feasibility threshold reported by study

Supplementary Table S11. Feasibility of physical activity interventions

| Author (year) | Recruitment | Retention | Participation | Adherence | | Adverse event |
| --- | --- | --- | --- | --- | --- | --- |
|  |  |  |  | **Intervention** | **Assessment** |  |
| Bogg 2015 [94] | 16/18, 89% | 100% | 63% | >50% of cohort  (60 mins/wk) |  | None reported |
| Chamorro-vina  2010 [89] | 9/14, 64.29% | 7/9, 77.8% | 92 (8) % | 69 (3 SD) % HRmax  (50-70%HRmax)  28 (3) mins |  | None reported |
| Huang 2023 [71] | 48/57, 84.2% | 44/48, (91.7%) | 70.7% | Mean RPE 12.6 (12-13.7)  Mean 24mins (30-40), 80%* |  | None reported |
| Morales 2020 [84] | 120/157, 82.8% | IG: 65/66, 98% CG: 53/54, 98% | median 11/15 sessions, 73.3%* | NR |  | None reported |
| Schecter 2023 [72] | 125/244, 51.2% | IG: 50/62, 80.6% CG: 50/63, 79.4% | Median number of yoga sessions 8 (IQR 6-10), 53.3%* | median duration of sessions 30 (IQR 20-40)  33% of sessions high intensity |  | None reported |
| Senn-Malashonak 2019 [73] | 70/77, 90.9% | 52/70, 74.3% | IG: 3.1/5 sessions/week, 62% CG: 2.9/5, 58% | RPE 13.7 (goal 12-14)  IG: 94.4 (63.3–100) % CG: 68.2 (5.8–100)% | Spiroergometric: 17/52, 32.7% | None reported |
| Jacobs 2021 [74] | 6/8, 75% | 4/6, 66.7% | IG: 22/36, 61.1% CG: 14/32, 43.6% | 1/6, 16.7%  (complete 9 sessions) |  | Multiple safety concerns |
| Rossi 2022 [85] | 49/49, 100% | 35/36, 97.2% | >60% in 72.4% of participants  >60% of sessions in at least >70% participants | 72.4% strengthening and stretching exercises, 20.7% for the aerobic exercises |  | None reported |

| Atkinson 2023 [101] | 21/28, 75% | 16/21, 76.19% | 83% |  |  | 49 events  2 moderate: pain  1 severe: wrist fracture (not during training), unlikely related to intervention. |
| --- | --- | --- | --- | --- | --- | --- |
| Bluth 2016 [135] | NR | NR | 1.8±1.6 (1-9) | NR | NR | NR |
| Caru 2020 [106] | 16/16, 100% | 100% | 9.6/12 (1.6 per week), 80% | Cardiovascular 50.1 (9.6) mins/week (30 mins)  Mean exercise and adapted functional activity 18.5 (4) mins/week (915 mins) | NR | None reported |
| Corr 2017 [87] | 20/25, 80% | 14/20, 70% | 85% (12/14) of participants completed >50% of PT sessions. 60% complete >50% sessions | NR | NR | None reported |
| Cox 2017  [56] | 85%  122/211, 57.8% | 94%  87.7% randomised  IG: 36/53, 67.9%  CG: 41/64, 64.1% | Visits  IG: APN 89.8%, PT 81.7%  PA logs not reported | NR | Accelerometer compliance 79-94%  Activity logs not reported | NR |
| Dalla Santa 2023 [103] | 16/17, 94.1% | 14/16, 87.5% | Mean 7.5/8 sessions | NR | 3/14 returned activity log. | None reported |
| Diorio 2015 [95] | 11/16, 68.8% | 10/11, 90.9% | Median 9/15 sessions, 60% (range 3-13),  60% of sessions (3x/week) in 70% of participants. | NR |  | None reported |
| Esbenshade 2014 [102] | 17/27, 63% | 12/17, 70.6% | 81.7 ± 7.2% | NR |  | NR |
| Fiuza-Luces 2017 [63] | 49/74, 66.2% | 100% | 68% (4%) completed sessions ^a^ |  |  | No major adverse events |
| Fukuhara 2020 [139] | NR | NR | NR | NR |  | NR |
| Gaser 2022 [64] | 41/70, 58.6% | Intervention: 20/21, 95.2% Control: 20/20, 100% | Intervention group: 65% of sessions Control group: 68% of sessions | Duration: 30.6 (4.2)  30 minutes | Accelerometer wear time 84.3%* | 3 minor events |
| Gohar 2011 [93] | 9/9, 100% | 9/9, 100% | 43/45, 98% | NR |  | None reported |
| Gotte 2018 [77] | Intervention: 24/25, 96% Control:  19/25, 73% | Intervention: 21/24, 87.5% Control: 100% | NR | Step goals 51.7% Active minutes 15.8% |  | NR |
| Govardhan 2019 [107] | 18/20, 90% | 100% | Median session attended 16/20, 80% *(12 sessions)* | NR |  | NR |
| Grimshaw 2024 [98] | 96% (21/22) (>75%) | 20/21, 95.2% (>90%) | 90% | Step goal: 85% | Fitbit inspire: Phase 1: 98%, phase 2: 79% (>90%),  Physical function assessment 74% | 19 mild |
| Hamari 2019 [65] | 36/47, 77% | 100% | NR | Median 30min/week, 14% (210mins) | Accelerometer  IG: 12/17  Activity diaries 12/17  Movement-ABC2 11/17  PedsQL 12/17 | None reported |
| Hartman 2009 [57] | 51/75, 68% | IG: 20/25, 80% CG: 21/26, 80.8% | 11% exercised daily, 37% >1 x a week, 16% 1x/week |  |  | IG: 8 fractures  CG: 4 fractures |
| Hayati 2019 [78] | NR | NR | NR | NR |  | NR |
| Hinds 2007 [66] | 29/30, 96.7% | 100% | 85.4% (70/82)  70% of sessions | NR |  | None reported |
| Hooke 2016  [105] | 17/28, 60.7% | 16/17, 94.1% | n/a | NR | Fitbit One compliance 92% | None reported |
| Hooke 2019 [88] | Standard care | n/a | 16% of EMR contained intervention related documentation |  |  | NR |
| Kaushal 2013 [58] | 36/36, 100% | NR | NR | NR |  | NR |
| Khodashenas 2017 [59] | NR | NR | NR | NR |  | NR |
| Kowaluk 2022 [67] | 27/36, 75% | 21/27, 77.8% | 100%^b^ | No Value reported |  | NR |
| Lam 2018 [68] | 70/89, 78.7% | Ig: 34/37, 91.9% CG: 29/33, 87.9% | n=37, 1024/1036, 98.8% | NR |  | None reported |
| Marchese 2004 [60] | 29/33, 87.9% | 28/29, 96.6% | NR | Ankle stretching 3/5 x/week, 60%  Strength 3/3x/ week, 100% | Activity logs not completed | None reported |
| Masoud 2023 [61] | 44/104, 42.3% | IG: 21/22, 95.5% CG: 23/23, 100% | 100% attendance to sessions | Mean exertional effort 56.5% (50-70%) |  | NR |
| Morales 2020 [76] | 169/183, 92.3% | 100% at post treatment follow up | Solids: 42 (19)/57 sessions, 73.7%  Leukaemia: 62 (12)/84 sessions, 73.8%* | NR |  | No major adverse events |
| Muller 2014 [79] | IG: 15/20, 75%  CG: 17/20, 85% | IG: 10/15, 66.7%  CG: 11/17, 64.7% | 77% | NR |  | None reported |
| Nielsen 2018 [96] | 75/78, 96.15% | 100% | 46% (IQR 33-58) |  | 69/78 (92%) (at least 1 physical function test) CPET 25%, HGS 81% Children with CNS tumours completed less tests (p<0.05). | NR |
| Nielsen 2020 [80] | IG: 120/128, 94% CG: 58/107, 54% | IG: 100% CG: 50/58, 86.2% | 68% or 3 sessions/5-day hospital admission | NR |  | 6 minor events |
| Fridh 2023 [104] | IG: 120/128, 93.8% CG: 50/107, 46.7% | Intervention: 75/120, 62.5% Control: 33/50, 66% | Median of 64% or 3 sessions per 5 days in hospital | NR |  | 6 minor events |
| Peterson 2022 | 18/20, 90% | 100% | n/a | n/a |  | NR |
| Orsey 2017 [109] | NR | 10/22, 45.5% | N/A | Duration: 80%* |  | NR |
| Oschwald 2023 [136] | 11/12, 91.7% | 8/11, 72.7% | 193/401 sessions, 48% | 171/193, 89% performed sessions per protocol |  | No serious adverse events. 21 sessions (11%) had to be stopped due to side effects related to WBV |
| Ouyang 2019 [81] | 143/147, 97.3% | IG: 57/76, 75% CG: 57/67, 85.1% | NR | NR |  | None reported |
| Platschek 2019 [90] | NR | 100% | Mean 10.67 (2.12SD) sessions | Mean duration 48.53 mins (14.85 SD) |  | None reported |
| Pouplier 2024 [142] | NR | NR | NR | NR |  | NR |
| San Juan 2007 [91, 97] | NR | 100% | >85% in all participants (>40/48 sessions attended) | NR |  | None reported |
| Saultier 2021 [69] | 80/177, 45.2% | IG: 37/41, 90.2% CG: 25/39, 64.1% | NR | NR |  | None reported |
| Sonoda 2024 [82] | 13/13, 100% | 12/13, 92.3% | 100% (80% of session) | NR |  | None reported |
| Stein 2019 [108] | 10/27, 37.0% | 8/10, 80% | 61/85, 72% sessions attended,  96% in person sessions attended, 61% skype sessions attended | Median duration 45 mins (IQR 40-45)  45 mins |  | None reported |
| Stossel 2019 [70] | 35/39, 89.7% | IG: 15/18, 83.3% CG: 16/17, 94.1% | 2.7 (1.2) sessions/week, 90% | Endurance: 10.4 (4.5) mins MI (15-20mins) Strength: 10.5 (3.3) mins MI (6-10mins) |  | No serious adverse events |
| Taguchi 2018 [137] | n/a | n/a | n/a | n/a |  | None reported |
| Tanner 2017 [100] | n/a as consent not required | n/a | 43/135, 32% completed the program and met discharge criteria | NR |  | NR |
| Thygeson 2010 [138] | 16/32, 50% | 15/16, 93.8% Parents 32/33, 97% | n/a | n/a |  | NR |
| Tsao 2022 [111] | N/A retrospective review | n/a retrospective review | 84% of planned PT sessions (8.39/10) | n/a |  | None reported |
| Wurz 2014 [92] | 11/24, 46% | 8/11, 73%  >70% | Mean 55% (13/24 sessions)  12/24 sessions | n/a |  | None reported |
| Waked 2018 [62] | 65/65, 100% | IG: 23/33, 69.7% CG: 23/32, 71.9% | NR | NR |  | NR |
| Winter 2013 [83] | IG: 28/31, 90.3% CG: 22/23, 95.7% | IG: 16/28, 57.1% CG: 15/22, 68.2% | 58.5%, mean 3.1 (1.1) sessions per 5.3 (1.7) days of inpatient stay (daily) | 35.6 ± 9.7 min/intervention (30-60mins) |  | None reported |
| Wright 2003 [86] | 40/40, 100% | NR | NR | NR |  | NR |
| Yeh 2011 [75] | 24/28, 85.7% | IG: 12/14, 85.7% CG: 10/10, 100% |  | Mean 76% [SD, 6.02] Completed 2/3 of intervention prescribed |  | NR |
| Zardo 2022 [140] | 213/255, 83.5% | 97/213, 45.5% | NR | NR |  | No major adverse events |

^a^ : Complete session defined as when ≥90% of the prescribed exercises were successfully performed; ^b^: participants excluded if 12 sessions were not completed; IG: Intervention group; CG: Control group; NA: Not applicable; NR: Not reported

Supplementary Table S12. Effect direction outcome domains

|  | Measure | Positive health impact | Change direction of negative health impact | Unable to determine |
| --- | --- | --- | --- | --- |
| Dietary intake | Diet quality (score) | Increase | Decrease |  |
|  | Fruit/Vegetables (serving/day, FFQ) | Increase | Decrease |  |
|  | Dairy (serving/day, FFQ) | Increase | Decrease |  |
|  | Sodium (mg/1000kcal, mg/day) | Decrease | Increase |  |
|  | Sugar (g/1000kcal, tsp, g/day, Sugar sweetened beverages | Decrease | Increase |  |
|  | Fiber (g/day, g/100kcal, | Increase | Decrease |  |
|  | Calorie intake (kcal/day) | Towards EER | Away from EER | Not compared to EER |
|  | Protein (g/day, g/kg, % kcal) | Towards requirements | Towards requirements | Not compared to requirements |
|  | Carbohydrates (g/day, g/kg, % kcal) | Towards requirements | Towards requirements | Not compared to requirements |
|  | Fat (g/day, g/kg, % kcal) | Towards requirements | Towards requirements | Not compared to requirements |
| Anthropometry & body composition | BMI for age/sex | Towards z-score of 0, 50th percentile | Away from z-score of 0, 50th percentile |  |
|  | Weight for age/sex | Towards z-score of 0, 50th percentile | Away from z-score of 0, 50th percentile |  |
|  | Height for age/sex | Towards z-score of 0, 50th percentile | Away from z-score of 0, 50th percentile |  |
|  | FFM (%, kg) | Increase | Decrease |  |
|  | FM (%, kg) | Decrease | Increase |  |
|  | BMD | Increase | Decrease |  |
|  | Nutrition status | Decrease in OW/OB | Increase in OW/OB |  |
| Exercise capacity | 6MWT (m) | Increase | Decrease |  |
|  | 9MWR (m) | Increase | Decrease |  |
|  | Vo2 Peak | Increase | Decrease |  |
|  | VO2 max | Increase | Decrease |  |
|  | VT | Increase | Decrease |  |
| Physical function | Timed up and go (s) | Decrease | Increase |  |
|  | Sit to stand (s) | Decrease | Increase |  |
|  | Timed rise from floor (s) | Decrease | Increase |  |
|  | Timed up and down stairs (s) | Decrease | Increase |  |
|  | Sit and reach (cm) | Increase | Decrease |  |
|  | Balance (s) | Increase | Decrease |  |
|  | Ankle dorsiflexion (degrees) | Increase | Decrease |  |
|  | BOT-2 | Increase | Decrease |  |
| Strength | HGS | Increase | Decrease |  |
|  | Repetition max (kg) | Increase | Decrease |  |
|  | Knee extension strength | Increase | Decrease |  |
| Physical activity | Steps | Increase | Decrease |  |
|  | Accelerometer (time) | Increase | Decrease |  |
|  | MVPA (min/day) | Increase | Decrease |  |
| Other | Fatigue | Decrease | Increase |  |
|  | Quality of life | Increase | Decrease |  |

References

141.	Lam KK, Li WH, Chung JO, Ho K, Xia W, Cheung AT, et al. (2020) Promoting physical activity among children with cancer through an integrated experiential training programme with coaching: A qualitative study. Patient Education and Counseling 103(6):1230-6.https://doi.org/10.1016/j.pec.2020.02.001

142.	Pouplier A, Fridh MK, Christensen J, Ruiz‐Molsgaard H, Høyer A, Schmidt‐Andersen P, et al. (2024) The potential of structured active play for social and personal development in preschoolers during cancer treatment: A qualitative RePlay study. Journal of Advanced Nursing 80(4):1607-18.https://doi.org/10.1111/jan.15923
